# Identification of Common Differentially Expressed Genes in Urinary Bladder Cancer

**DOI:** 10.1371/journal.pone.0018135

**Published:** 2011-04-04

**Authors:** Apostolos Zaravinos, George I. Lambrou, Ioannis Boulalas, Dimitris Delakas, Demetrios A. Spandidos

**Affiliations:** 1 Laboratory of Virology, Medical School, University of Crete, Crete, Greece; 2 Choremeio Research Laboratory, First Department of Pediatrics, University of Athens, Athens, Greece; 3 Department of Urology, Asklipieio General Hospital, Athens, Greece; Texas A&M University, United States of America

## Abstract

**Background:**

Current diagnosis and treatment of urinary bladder cancer (BC) has shown great progress with the utilization of microarrays.

**Purpose:**

Our goal was to identify common differentially expressed (DE) genes among clinically relevant subclasses of BC using microarrays.

**Methodology/Principal Findings:**

BC samples and controls, both experimental and publicly available datasets, were analyzed by whole genome microarrays. We grouped the samples according to their histology and defined the DE genes in each sample individually, as well as in each tumor group. A dual analysis strategy was followed. First, experimental samples were analyzed and conclusions were formulated; and second, experimental sets were combined with publicly available microarray datasets and were further analyzed in search of common DE genes. The experimental dataset identified 831 genes that were DE in all tumor samples, simultaneously. Moreover, 33 genes were up-regulated and 85 genes were down-regulated in all 10 BC samples compared to the 5 normal tissues, simultaneously. Hierarchical clustering partitioned tumor groups in accordance to their histology. K-means clustering of all genes and all samples, as well as clustering of tumor groups, presented 49 clusters. K-means clustering of common DE genes in all samples revealed 24 clusters. Genes manifested various differential patterns of expression, based on PCA. YY1 and NFκB were among the most common transcription factors that regulated the expression of the identified DE genes. Chromosome 1 contained 32 DE genes, followed by chromosomes 2 and 11, which contained 25 and 23 DE genes, respectively. Chromosome 21 had the least number of DE genes. GO analysis revealed the prevalence of transport and binding genes in the common down-regulated DE genes; the prevalence of RNA metabolism and processing genes in the up-regulated DE genes; as well as the prevalence of genes responsible for cell communication and signal transduction in the DE genes that were down-regulated in T1-Grade III tumors and up-regulated in T2/T3-Grade III tumors. Combination of samples from all microarray platforms revealed 17 common DE genes, (BMP4, CRYGD, DBH, GJB1, KRT83, MPZ, NHLH1, TACR3, ACTC1, MFAP4, SPARCL1, TAGLN, TPM2, CDC20, LHCGR, TM9SF1 and HCCS) 4 of which participate in numerous pathways.

**Conclusions/Significance:**

The identification of the common DE genes among BC samples of different histology can provide further insight into the discovery of new putative markers.

## Introduction

Cancer of the urinary bladder (BC) is the fifth most common cancer in men. The peak prevalence of the disease is among patients 60–70 years of age. BC is curable if diagnosed during the early stages of the disease. Tumors of the urinary bladder develop via two distinct but somewhat overlapping pathways: the papillary and non-papillary. Approximately 80% of BCs consist of superficial exophytic papillary lesions that originate from urothelial hyperplasia. These typically low-grade papillary tumors may recur, but rarely invade the bladder wall or metastasize. The remaining 15–20% of tumors represent high-grade solid non-papillary BCs that arise from high-grade intraurothelial neoplasia. These tumors aggressively invade the bladder wall and have a high propensity for distant metastasis [1].

Parallel gene-expression monitoring is a powerful tool for analyzing relationships among bladder tumors, discovering new tumor subgroups, assigning tumors to pre-defined classes, identifying co-regulated or tumor stage-specific genes and predicting disease outcome [2], [3], [4], [5], [6], [7], [8]. To date, much effort has been spent in order to identify genes that display differential expression (DE) between various tumor types vs. other tissue groups, such as phenotypically normal tissue. However, the reported DE genes vary among different study groups, depending on the microarray-based methodology and the number of cases. One intriguing issue that has not as yet been contemplated involves possible data that can be gathered regarding genes that are differentially expressed simultaneously in all study tumor cases.

In the present study, we performed cDNA microarray analysis, comprising in-house experimental as well as publicly available data, to analyze the gene expression profile of BC and to determine the DE genes between cancer and healthy tissue. The detection of DE genes was performed in each sample individually, as well as in each tumor group, as defined by histological examination and reported data. Data were clustered with different algorithms, and functions of the known DE genes were further defined by Gene Ontology. Furthermore, we searched not only for differences among tumor types, but rather for similarities. The reason for this approach was that, although different tumor types are expected to have differences in their expression profiles even between individuals with the same tumor subtype, we hypothesized that tumors possess similar characteristics that may eventually lead to knowledge of the etiologies of carcinogenesis. In a significant work by *Goldstein et al.* an attempt to detect a common cell of origin for prostate cancers was reported. They implied that, despite the differences that tumor cells do share, there is a possibility of a common origin [9]. In the same direction we attempted to identify common gene expression profiles among different tumor tissues. At this point we should mention that gene expression, particularly from tumor biopsies, represents literally a “snap-shot” of the state-space of the otherwise dynamic behavior of the disease. Yet, this “snap-shot” might be adequate in order to obtain useful information on the dynamics of the system of study. Particularly in the case of tumors, it is the only tool we have in order to extract information at the *ex vivo* level.

The present data support the value of microarray-based gene expression signatures as these identify clinically important cellular properties.

## Materials and Methods

### Tumor Tissue Sampling and Surgical Procedure

Ten urinary bladder cancer specimens from patients with newly diagnosed BCs undergoing transurethral bladder tumor resection at the Department of Urology, “Asklipieio” General Hospital, Athens, as well as five normal tissue samples, were acquired after the amount of tissue necessary for routine pathology examination had been removed. The patients studied were of advanced age (73.9±12.0 years). The majority (6/10, 60%) were smokers or former-smokers, whereas four (40%) were characterized by some level of occupational exposure to agents associated with BC (paints, chemicals, etc.). All tumor specimens were classified and graded by the same pathologist. Histological grading was performed using both the 1973 World Health Organization (WHO) and the 2004 WHO/International Society of Urologic Pathology (ISUP) classifications [1]. Tumor stage was assessed according to the 2002 American Joint Committee on Cancer staging system [10]. Written informed consent was obtained from all patients included in this study. The study protocol was approved by the Ethics Committee of the University of Crete. Eligibility criteria used were electively dissected primary BCs and the availability of DNA from normal and tumor tissue for biomolecular analyses. Exclusion criteria were a history of previous neoplasms and chemotherapy or radiation therapy prior to surgery.

Tissue samples were obtained at surgery from the tumor and the following three grossly normal selected sites (cold cup biopsies): posterior wall, trigone, and area adjacent to the tumor. Parts of the dissected normal samples were sent for histopathological analysis. Tumor and normal tissues were frozen immediately in liquid nitrogen, transported and stored at −80°C until DNA extraction.

Patients with non-muscle-invasive BCs were followed up with periodical cystoscopic examinations and intravesical treatment as indicated. Patients with invasive BCs were offered radical cystectomy with or without systemic chemotherapy.

### Immunohistochemistry

Sections, 3 mm thick, of formalin-fixed, paraffin-embedded tissue were cut and placed on slides coated with 3-aminopropyltriethoxysilone. Slides were dried at 56°C for 1 h before immunohistochemical staining. Tissue sections were deparaffinized in xylene before rehydration in graded alcohols, and endogenous peroxidase activity was blocked by treatment with 3% H_2_O_2_ at room temperature for 15 min. Antigen unmasking was performed by 30 min of incubation at 80°C in 10 mM trisodium citrate (pH 6.1). Immunostaining and revelation were performed on a Dako automate. Slides were incubated at room temperature with primary polyclonal goat antibodies against anti-ErbB2 (1∶800; Dako), cyclin D1 (1∶100; SP4; Epitomics), monoclonal antibody against anti-p53 (1∶250; E26; Epitomics) and monoclonal antibody against anti-Ki-67 (1∶100; SP6; Epitomics). Epitopes of the primary antibody were localized by immunoperoxidase technique using the secondary antibody avidin-biotin complex and peroxidase substrate kit (kit 5001, Dako), according to the manufacturer's protocol. The sections were then treated with Chromagen 30–30 diaminobenzidene tetrahydrochloride to identify sites of immunoprecipitation by light microscopy. Finally, sections were washed, counterstained with hematoxylin, and mounted under cover slips. No specific staining was observed when the primary antibody was omitted from the protocol (negative control). Specificity of the immunostaining was additionally controlled by simultaneous staining of breast cancer samples with known ErbB2, cyclin D1, p53 and Ki67 expression patterns. An experienced pathologist scored the staining intensity at four levels (negative, weak, moderate and strong), considering both color intensity and number of stained cells.

### Microarray Experimentation and Inclusion of Publicly available Microarray Datasets

Oligos microarray chips (∼57 k genes) were obtained from GE HealthCare (IL) and AppliedMicroarrays (MA) (former Amersham Biosciences) (CodeLink 57 k Human Whole Genome) [11], [12], [13]. Hybridization was performed with the CodeLink RNA amplification and Labeling kit as described by the manufacturer, utilizing the Cy5 fluorescent dye. Slides were scanned with a microarray scanner (ScanArray 4000XL). Images were generated with ScanArray microarray acquisition software (GSI Lumonics, USA). cRNAs from three experimental setups were used in single experiments with internal spikes as controls. The experimental setups consisted of 10 urinary BC samples of different histology (see Tissue Sampling and Surgical Procedure section) and 5 control samples. The scanned images were further processed with the CodeLink Expression Analysis Software v5.0 from Amersham Biosciences (presently GE Health Care Inc.). The experimental setup was analyzed based on the reference-design as described previously [14], [15], [16] as presented in **Figure S1A**. All tumor samples were compared against the mean value of the control samples. Raw microarray data are available at the GEO microarray database. All microarray data are MIAME compliant.

In order to expand the number of BC samples under investigation, we included the following publicly available microarray datasets in our analysis: 1) GSE89 dataset (GDS183) [17], comprised of 40 BC samples; 2) GSE3167 dataset (GDS1479) [18], comprised of 60 samples (9 controls and 51 BC samples); 3) GSE7476 dataset [19], composed of 12 samples (3 controls and 9 BC samples) and 4) GSE12630 dataset [20], comprised of 19 BC samples. In total, our pooled microarray analysis was composed of 17 control samples (n = 5, for the CodeLink platform; and n = 12, for the remaining microarray platforms) and 129 BC samples (n = 10, for the CodeLink platform; and n = 119, for the remaining microarray platforms). Public data were used in their available normalized form, since background correction and normalization had already been performed.

### Microarray Data processing

#### Microarray Data Filtering and Background Correction of the CodeLink Platform

Filtering is performed based on the signal intensity and on the criterion of whether this signal is above a certain level. In our analysis, filtering was performed using the equation: 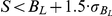, where S is the measured signal intensity, B_L_ is the local background measured and σ_BL_ is the standard deviation of the local background. Signals with intensity lower than the above measured, obtained a flag. Background correction was performed by subtracting the median global background from the median local background from the signal intensity. A threshold of 2 was set as cut-off, meaning that spot intensity for at least one channel should be twice as much as that of the background.

#### Microarray Data Normalization of the CodeLink Platform

Microarray data were normalized by the default procedure of the CodeLink software, i.e. spot intensities were divided by the global median (global median normalization) [13]. Normalized data were extracted, pre-processed and sorted with Microsoft Excel ®. For further data analysis the Matlab ® (The Mathworks Inc.) computing environment was used. Data were examined for their distribution pattern for further choice of the statistical test method.

#### Microarray Data Cross-Normalization

Microarray data were cross-normalized, using a quantile algorithm, in order to account for the bias that was included due to experimentation, different platforms and different sampling (**Figure S2**). Normalization of cross-platform data has been previously described [21], [22], [23] with very good correlations and consistency between the CodeLink and Affymetrix platforms [24], [25].

### Creating a Common Gene List and BC Groups

In order to ensure that the results of our analysis were comparable, we created a common gene list among all microarray platforms. For this purpose, the NCBI Gene ID number was used as a common reference. After comparing the DE genes among all datasets, only those present in all platforms were selected for further analysis. In total, this filtering approach yielded a gene set of 11,837 unique records, simultaneously present in all microarray platforms. Datasets were used as individual samples as well as in tumor groups. The group distribution is presented in Table S1.

### Microarray Data Statistics for the CodeLink Platform and the Publicly available Microarray Datasets

In regards to the CodeLink platform, our approach consisted of the following methodology. Each gene was tested for its significance in differential expression using a z-test. Genes were considered to be significantly differentially expressed if they obtained a p-value<0.05. Comparisons were made both among experiments as well as within experiments. Set manipulation was then used in order to discover further subsets that would characterize, if possible, all tumor samples. For further analyses we used the genes that were differentially expressed among tumor samples.

Regarding the comparison among genes of all of the available microarray datasets, we used the following methodology:

- First, we searched for differences, comparing all control samples (considered as one group), against all tumor samples (considered as another group), using a two-tailed two-sample T-test. Since these groups contained samples which varied in ethnicity and tumor grade, we controlled all bias by comparing them as unified groups.
- Second, we separated samples into groups (11 groups in total) (**Table S1**), and each group was compared against all control samples, using a two-tailed two-sample T-test.
- Third, we compared samples individually for significant genes among each experiment, using a two-tailed z-test, which is referred to as “intra-experimental”. This type of comparison had a particularity. Since expression of the genes was compared to the mean of the gene expression within the same experiment, the DE genes would signify the difference that each tissue sample exhibited in comparison with the normal tissues. This means that the common genes among them would be those genes that are common to the tumor tissue.
- We compared samples individually for significant genes i.e. gene ratios, among experimental setups, using a two-tailed z-test, which we refer to as “inter-experimental”. In other words, we searched for genes that exhibited different expression from one sample to the next but not against the control samples. Interestingly, the significant genes derived from these included genes that were not DE. In other words, these genes were identified among those whose expression remained the same across all samples.

In order to identify the differentially expressed genes, we used two methods. Genes were considered to be significantly differentially expressed if they obtained a p-value<0.05. Comparisons were made both among experiments as well as within experiments. Set manipulation was then used in order to discover further subsets that would characterize, if possible, all tumor samples. For further analyses we used the genes that were differentially expressed among tumor samples, on a need-to-use basis. In the case where sample groups were compared, the mean of each gene was taken against the mean of all control samples. In the case of individual comparisons of samples, gene ratios were calculated against the mean of all control samples.

### False Discovery Rate (FDR)

The False Discovery Rate was calculated as previously described [26], [27], [28].

### Clustering Analysis

Clustering analysis was performed with the k-means algorithm. In total, 81 clusters of the complete dataset of the CodeLink platform and 100 clusters for all available datasets, were formed and DE genes were further classified using two-way (genes-against-samples) average-linkage hierarchical clustering with Euclidian distance [29]. Clustering analysis and chromosome mapping were in part performed with Genesis 1.7.2 (Technische Universitaet-Graz, Austria) using Pearson's correlation (*r*) and Spearman's rank order correlation (*ρ*) [30], [31], [32].

### TFBM Analysis

In order to identify the transcription factors driving the observed changes in the gene expression, we investigated the Transcription Factor Binding Motifs (TFBMs) in the Transcription Element Listening System Database (TELiS) (www.telis.ucla.edu) [33]. The TRANSFAC transcription factor database was used for the identification of gene transcription factor binding sites [34].

### Chromosome Mapping

Chromosome mapping appears to be a promising method for identifying patterns among genes. The main idea reported initially by *Cohen et al*. is to map genes on chromosomal regions and in this way if correlations do exist they appear through the location of genes on chromosomal regions, since consecutive genes are often similarly expressed [30]. For chromosome mapping analysis, we used the Gene Ontology Tree Machine, WebGestalt web-tool (Vanderbilt University, The Netherlands, http://bioinfo.vanderbilt.edu/gotm/) [35] and the Matlab ® (The Mathworks Inc.) computing environment.

### Gene Ontology (GO) Analysis

Gene Ontology (GO) analysis was initially performed using the eGOn online tool for Gene Ontology (The Norwegian University of Science and Technology, Trondheim, Norway, http://www.genetools.microarray.ntnu.no/egon/) in order to find missing gene symbols [36]. WebGestalt web-tool (Vanderbilt University, The Netherlands, http://bioinfo.vanderbilt.edu/gotm/) [35], [37] was used for gene function classifications. Relations of the differentially expressed genes and the transcription factor binding motifs were further investigated using the Pubgene Ontology Database (www.pubgene.org). Gene definitions and functions were based on the National Institute of Health databases (http://www.ncbi.nlm.nih.gov/sites/entrez/).

### GEO accession numbers

Array data were deposited at the Gene Expression Omnibus (National Center for Biotechnology Information) with accession numbers GSM678186 through GSM678385 (http://www.ncbi.nlm.nih.gov/geo/query/acc.cgi?acc=GSE27448).

## Results

### Immunohistochemistry

**Figure 1:**
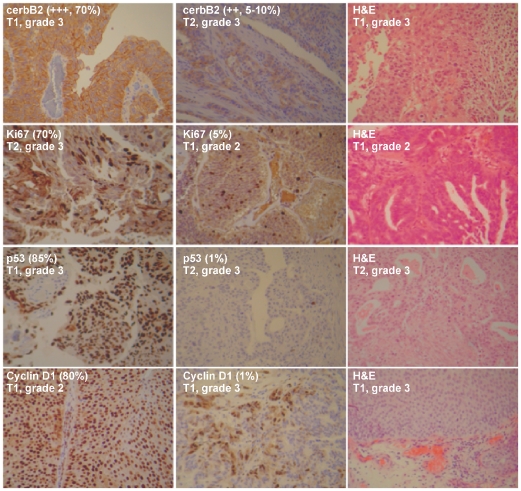
T2-Grade III tumors exhibited the strongest immunostaining for anti-cerbB2 (+++, >70%), anti-Ki67 (>70%) and anti-p53 (85%). On the other hand, T1-Grade II tumors showed intense staining for anti-Cyclin D1 (80%), whereas T1-Grade III tumors exhibited weak immunostaining. Representative H&E slides denote the histology of T1-Grade II, T1-Grade III and T2-Grade III tumors.

Tumor samples were stained with antibodies for ErbB2, cyclin D1, p53 and Ki-67. If present, anti-ErbB2 staining in tumor samples is a membrane staining, diffuse in the urothelium (**Figure 1**). All TCC samples (100%) showed moderate/strong (++, +++) immunostaining, whereas no TCC sample showed no/weak immunostaining (0, +). Thresholds for high labeling indices were set for Ki-67 at ≥10% positive tumor nuclei and for p53 at 10 and 20%. T1/2-Grade III tumors exhibited the strongest immunostaining (+++, >70%). T1-Grade I/II tumors showed weak staining for anti-Ki-67 (40% and 7.5%, respectively), whereas T1/2-Grade III tumors exhibited the strongest immunostaining (54%). Similarly, T1-Grade I/II tumors showed weak staining for anti-p53 (10% and 35%, respectively), whereas T1/2-Grade III tumors exhibited the strongest immunostaining (53%). On the other hand, T1-Grade I/II tumors showed intense staining for anti-Cyclin D1 (80% and 80%, respectively), whereas T1/2-Grade III tumors exhibited weak immunostaining (31.8%).

### CodeLink Platform Data Distribution and Analysis

Microarray data were investigated for their normal distribution property. The normalized data followed a normal distribution as presented in **Figure S1B** and **S1C**. Z-test statistics were applied on the data, both on individual samples as well as on tumor groups. In the case of tumor groups, genes that obtained a p-value<0.05 were considered differentially expressed. In **Figure S3A–C** the distribution of the p-values is presented. FDR was calculated as previously reported [27], [28]. FDR was calculated to be 9.3% for a p-value<0.05 for tumors of the T1-Grade II group, 8.6% for p-value<0.05 for tumors of the T1-Grade III group and 11.03% for p-value<0.05 for tumors of the T2 and T3-Grade III group (**Figure S3D–F**). The same procedure was followed for each sample individually. Genes were plotted in box-plots in order to examine the expression distributions in further detail. Box-plots of tumor groups as well as those for individual samples are depicted in **Figure S4A** and **Figure S4B**, respectively.

### Analysis of Common Differentially Expressed Genes: CodeLink Platform

In order to identify gene expression patterns in urinary BC, we further analyzed our microarray data in such a way that common patterns of expression among the different samples were identified. We focused our analysis, not only on the differences between tumor samples, but also on their similarities. It was not surprising that such similarities did exist. Intersections of individual tumor samples revealed 831 genes that were simultaneously differentially expressed in all of the tumor samples (either up-, or down-regulated). Of these DE genes, we identified 33 genes with simultaneous increased expression and 85 genes with simultaneous decreased expression in all BC samples, compared to normal tissue.

Among the up-regulated genes, those presenting the highest fold expression (mean±SD) were: hypoxia-inducible protein 2 (HIG2; NM_013332.1) (2.70±0.54); APC11 anaphase promoting complex subunit 11 (ANAPC11; NM_001002245.1) (2.50±0.60); zo54e12s1 Stratagene pancreas (#937208) cDNA clone IMAGE:590734 3′ similar to TR:G1022718 G1022718 NUCLEAR RECEPTOR CO-REPRESSOR (NCOR1; AA156336.1) (2.01±1.13); UI-1-BB1p-atp-e-01-0-UIs1 NCI_CGAP_Pl6 cDNA clone UI-1-BB1p-atp-e-01-0-UI 3′, (BU754189; BU754189.1) (1.89±0.56). Similarly, the genes that exhibited the lowest fold expression rates (mean±SD) were: tn52a12.x1 NCI_CGAP_Kid11 cDNA clone IMAGE:2171998 3′ similar to contains PTR5.b2 MER22 repetitive element (AI565993; AI565993.1) (−3.13±0.66); zx51b02r1 Soares_testis_NHT cDNA clone IMAGE:795723 5′ (AA461577; AA461577.1) (−2.75±0.86); lactotransferrin (LTF) (ANKRD29; NM_173505.2) (−2.28±1.17); HUMNK566 Human epidermal keratinocyte cDNA clone 566 (ODZ2; D29453.1) (−2.15±1.02); tumor necrosis factor receptor superfamily, member 17 (TNFRSF17) (TNFRSF17; NM_001192.2) (−2.07±0.73); and skeletal muscle LIM-protein FHL1 mRNA (FHL1; U60115.1) (−2.06±0.87).

Regarding the three tumor groups (T1-Grade II, T1-Grade III and T2/T3-Grade III), our analysis did not show a subset of genes common to all groups. Therefore, we investigated the common DE genes between pairs of tumor groups (**Tables S2, S3, S4**). DE genes common to all individuals regardless of tumor group, were further clustered using hierarchical clustering with Euclidean distance. Groups of common genes in several combinations are presented in **Table S5**.

#### Hierarchical Clustering with Euclidean Distance: CodeLink Platform

**Figure 2:**
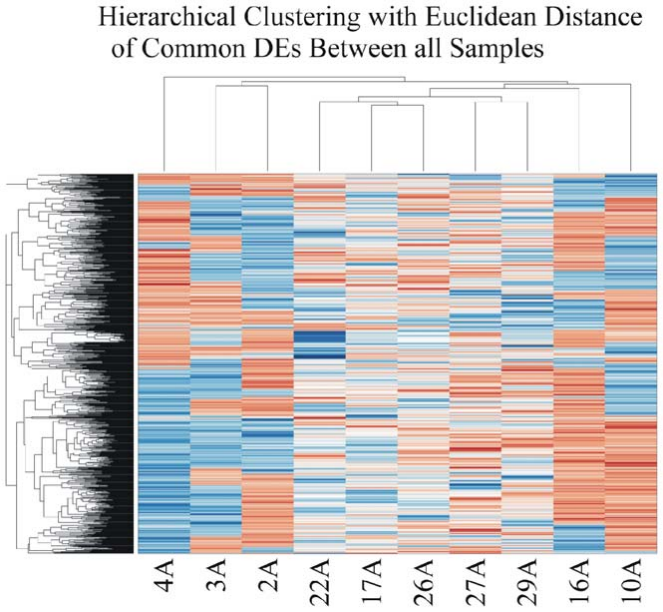
Hierarchical clustering with Euclidean distance revealed groups of genes of common and differential expression.

We performed two-way average-linkage hierarchical clustering with Euclidian distance for ∼57 k genes. A detailed view of the sample cluster dendrogram is displayed in **Figure 2**. We partitioned the tumors into two main groups and several subgroups based on the differential expression of their genome. The first branch contained a T1-Grade II tumor and the other contained tumors of T1-3-Grade II/III, which were further clustered into additional subgroups.

#### K-means clustering: CodeLink Platform

**Figure 3:**
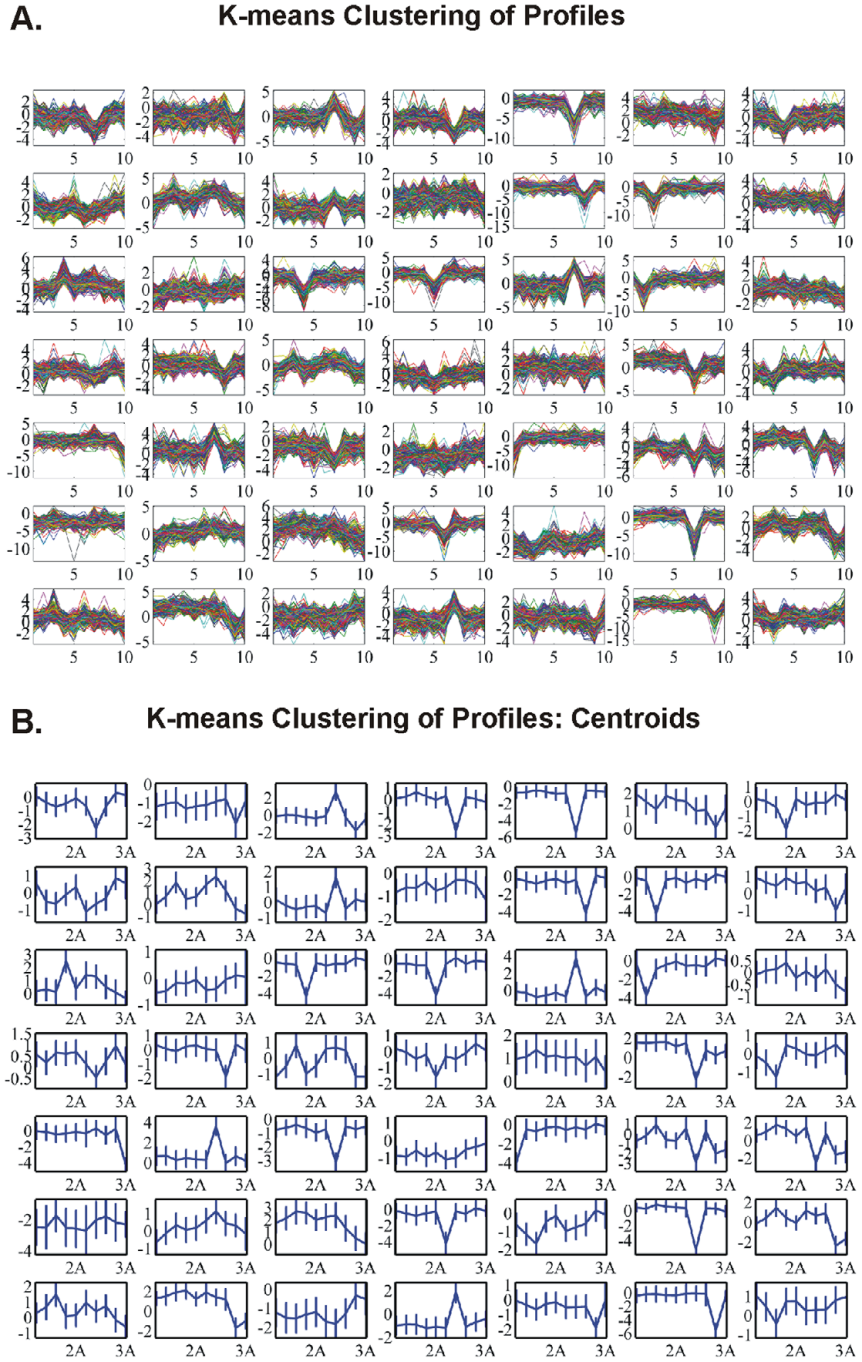
K-means clustering of all genes and all individual samples. K-means cluster gave some distinct patterns among samples, such as in clusters 1, 3, 4, 5, etc.

**Figure 4:**
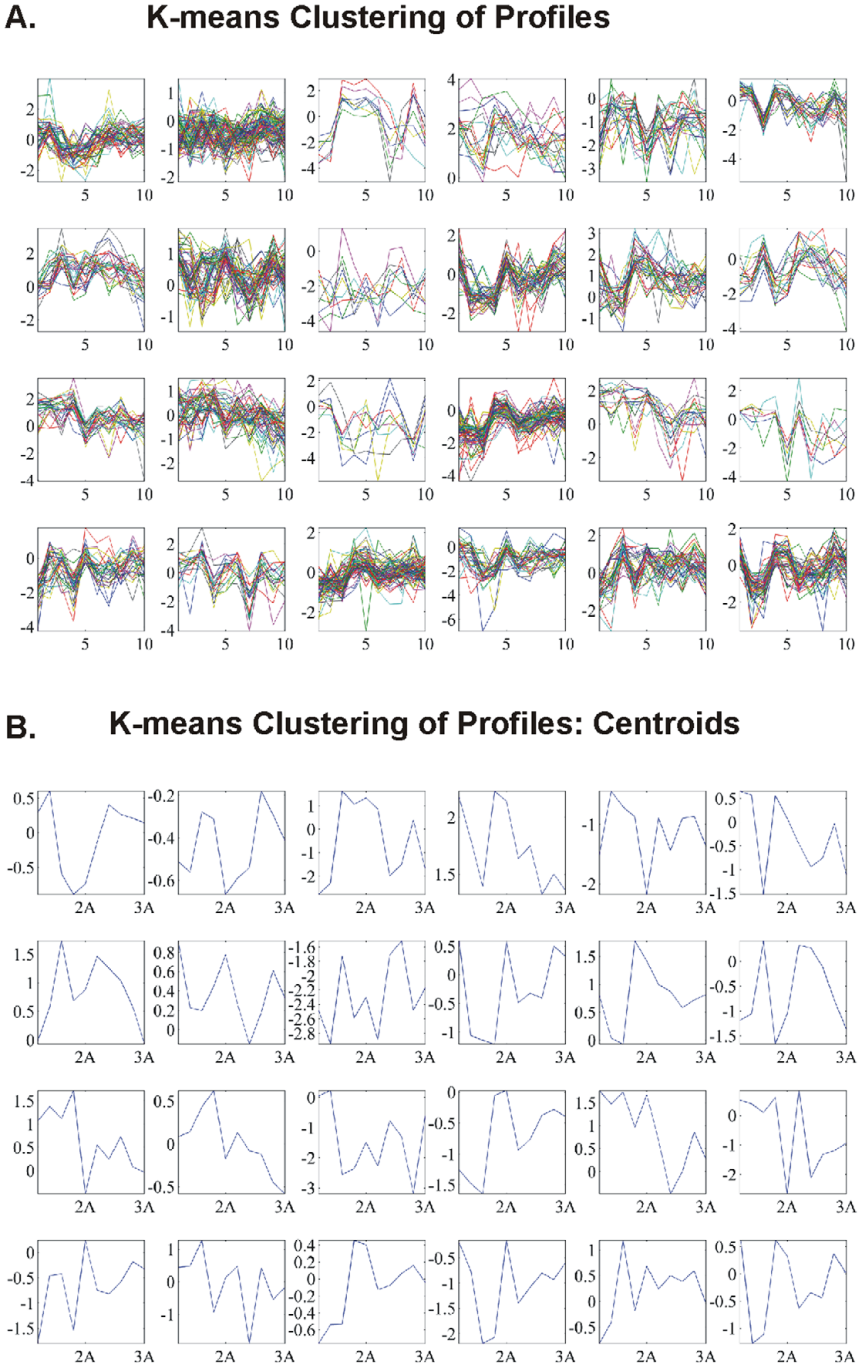
K-means clustering of common DE genes among all samples. Clusters (A) and centroids (B) are presented where no clear distinction can be made between individual samples.

**Figure 5:**
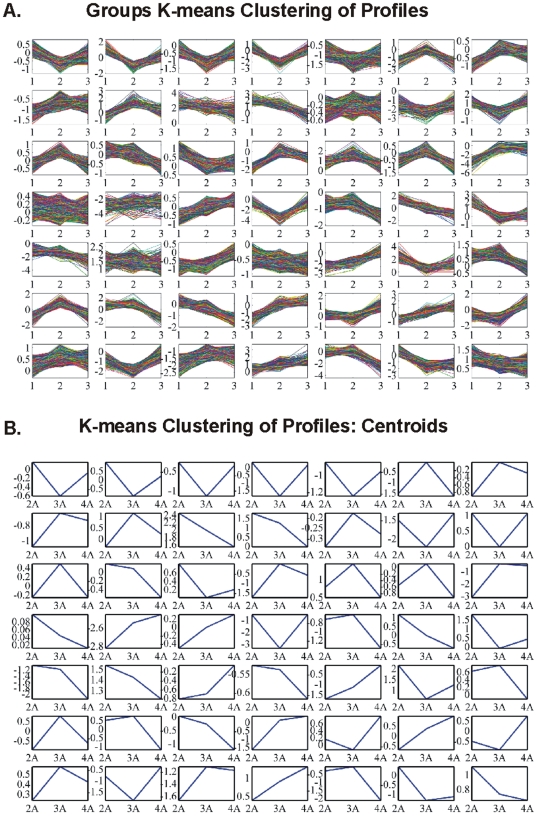
K-means clusters (A) and respective centroids (B) of tumor groups: T1-Grade II, T1-Grade III and T2/T3-Grade III.

K-means clustering algorithm is another way of classifying data in order to find patterns of expression. Our analysis was organized as follows:

- k-means of all genes and of all samples. The result of k-means clustering of all genes and all samples is presented in **Figure 3A**. We clustered data in 49 clusters along with their centroids (**Figure 3B**). For each cluster group there were several genes that characterized the cluster i.e. characterized the sample that the genes belonged to.
- k-means of common DE genes in all samples. The common DE genes among all samples were furthered clustered (**Figure 4A and 4B**).
- k-means of tumor groups. To conclude the analysis based on k-means clustering the tumor groups were analyzed as defined above. The results are presented in **Figure 5A and 5B** for cluster and centroids, respectively. Clusters of tumor groups revealed both differences as well as similarities between the different groups. For example, certain gene categories revealed constitutive down-regulation in one tumor group vs. the group of higher stage/grade, whereas other gene categories exhibited constitutive up-regulation between the corresponding groups (clusters 11, 24, 27, 30, 33, 41, 46). At the same time several clusters included genes that remained unchanged between tumor groups (clusters 21, 26, 35, 37, 45, 47, 48).

#### Principal Component Analysis (PCA): CodeLink Platform

**Figure 6:**
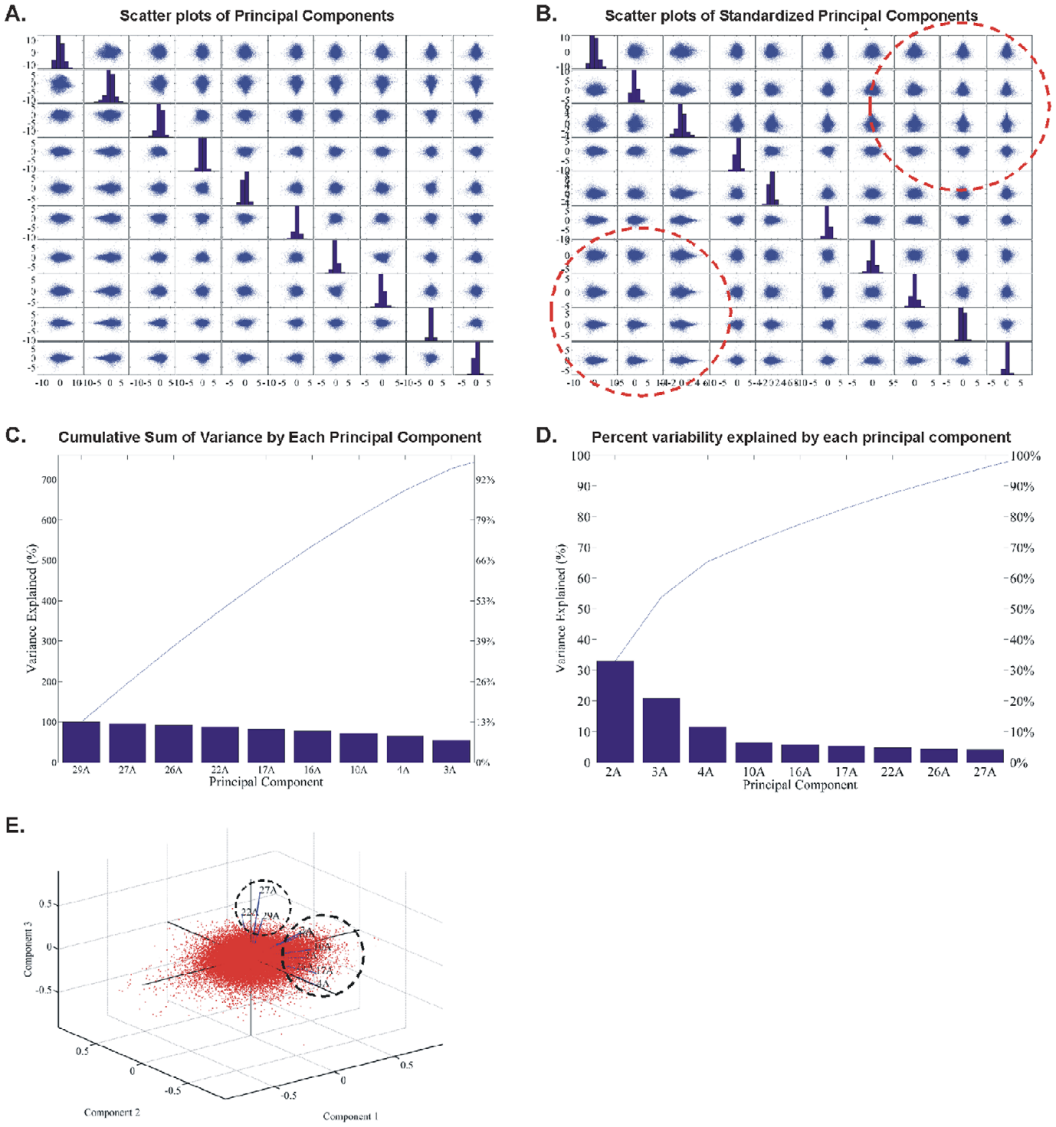
PCA analysis of genes was performed in order to find further patterns in the expression data. The first step to perform the present analysis with all genes and in all samples was to plot scatter plots of all combinations of principal components (A, B). Genes manifested several patterns as it is seen in the circled areas in B. Then, samples were examined for the percentage of variance they attributed to the principal components (C, D). Finally, a biplot was drawn on order to examine sample classification with respect total gene expression (E). As it is presented in the circled areas in E, samples were grouped into two main categories: samples 22A (pT2- pT3-Grade III), 27A (pT1-Grade III) and 29A (pT2- pT3-Grade III).

**Figure 7:**
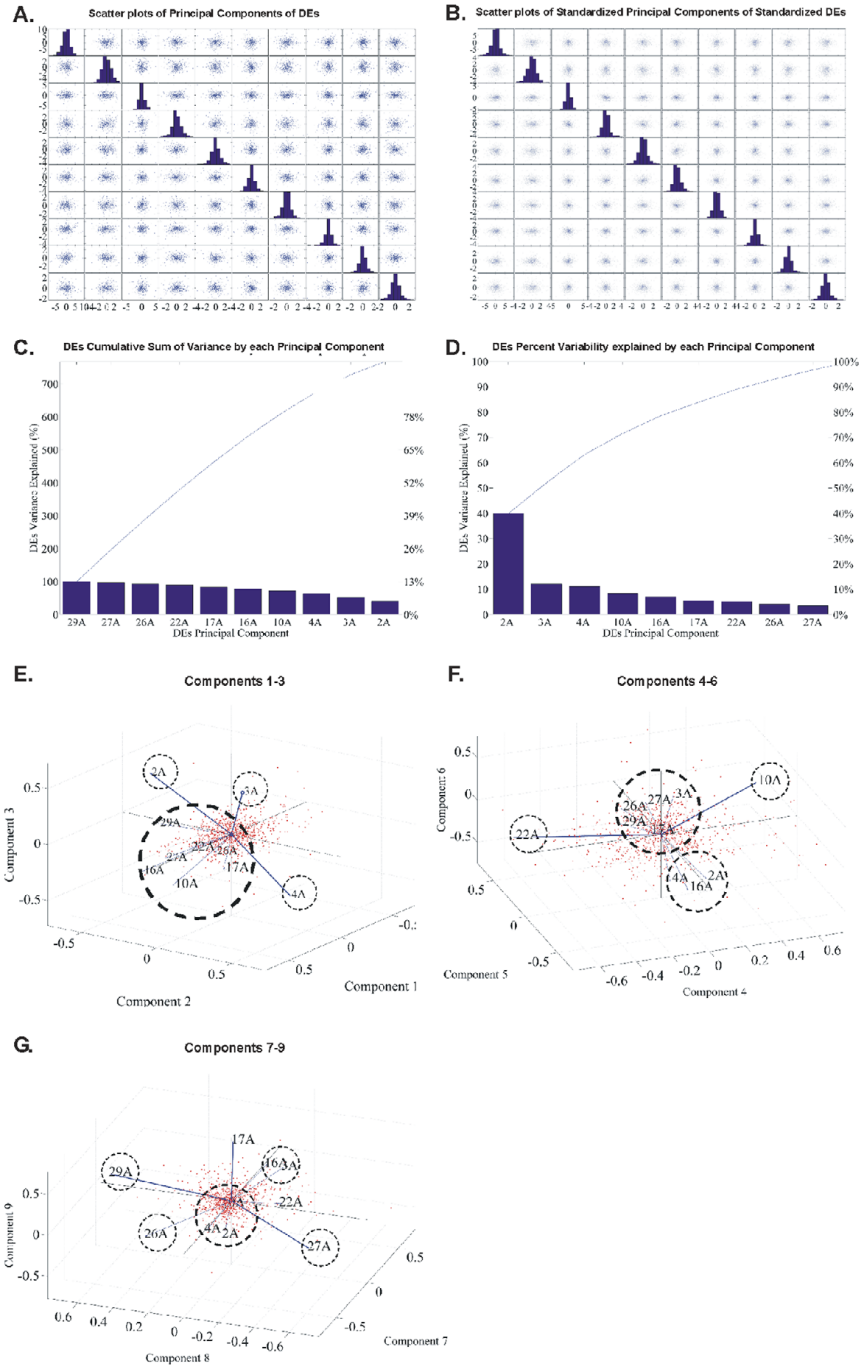
PCA analysis of common DE genes. Scatter plots of principal components (A, B) are presented. Sample 2A attributes the observed variance (D). Plotting of the components showed different groupings among samples as it is shown in E, F, G.

**Figure 8:**
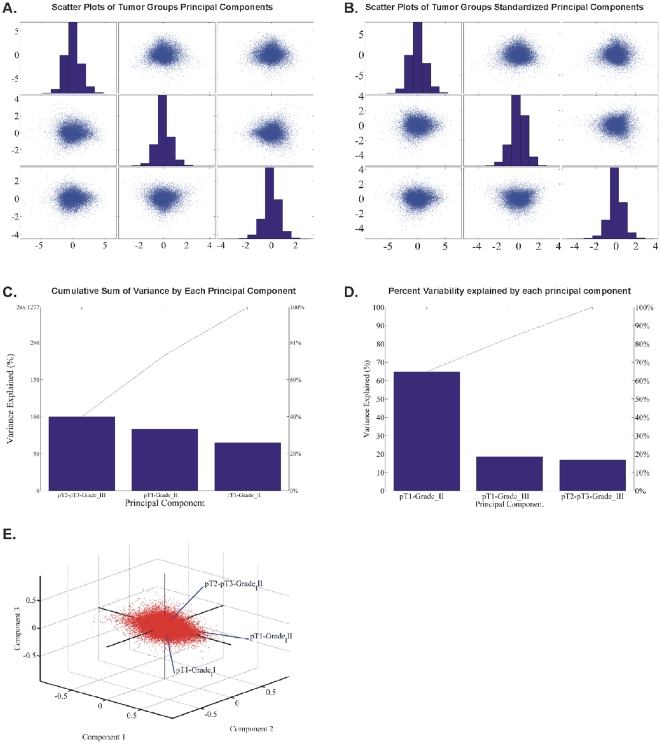
PCA analysis of tumor groups. Scatter plots of principal components are presented (A, B), observed variance (C, D) and biplot classification of tumor groups with respect to principal components (E).

- PCA of all genes in all samples. PCA analysis of our data was further carried out in order to search for other potential patterns among the genes. The initial analysis was performed with genes that were commonly DE among all samples. Calculated principal components were plotted against each other in scatter plots as presented in **Figure 6**. PCA analysis of the genes was performed in order to find further patterns in the expression data. To perform the present analysis with all genes and in all samples, the initial step was to plot scatter plots of all combinations of the principal components (**Figure 6A, B**). The genes manifested several patterns as noted in the circled areas in **Figure 6B**. Then, samples were examined for the percentage of variance they attributed to the principal components (**Figure 6C, D**). Finally, a biplot was constructed in order to examine sample classification with respect to total gene expression (**Figure 6E**). As presented in the circled areas in **Figure 6E**, samples were grouped into two main categories: samples 22A (T2/T3-Grade III), 27A (T1-Grade III) and 29A (T2/T3-Grade III) on one hand, and the rest of the samples were grouped together. In order to further resolve for differences based on PCA analysis we compared gene expression as follows.
- PCA of common DE genes in all samples. PCA analysis of the common DE genes is presented in **Figure 7**. Grouping of the samples based on principal components showed different classifications. Tumor samples 2A, 3A and 4A were grouped distinctively as per the first three principal components (**Figure 7E**). These three samples were grouped together when the complete data set was considered. Resolving this result with the common DE genes showed a difference between tumor groups. Similarly, several different groupings were obtained by PCA analysis of the common DE genes among all samples, such as among tumor samples 22A, 10A, samples 2A, 4A, 16A that formed a separate group and samples 3A, 17A, 26A, 27A, 29A that formed another one (**Figure 7F**). Similarly, plotting of the components grouped samples 3A and 16A, as well as samples 2A, 4A and 10A in a separate group.
- PCA of the tumor groups. PCA analysis of tumor groups showed a distinct separation among the three groups (T1-Grade II, T1-Grade III and T2/T3-Grade III) (**Figure 8**).

#### Transcription Factor Binding Motif (TFBM) Analysis: CodeLink Platform

One of the main parts of our data analysis included the determination of the over-represented TFBMs in the promoters of the DE genes. This provides inferences about which transcription factors are active. We searched for TFBMs in all combinations of gene expression as presented in **Table 1** and the results are presented in **Table S4**. Attention was focused on two transcription factors, YY1 and NFκB. YY1 appeared to commonly regulate the expression of down-regulated genes in T1-Grade II and simultaneously in T1-Grade III. NFκB, on the other hand, appeared to regulate common DE genes between tumors of T1-Grade III and those of T2/T3-Grade III. In particular, it appeared that the p65 subunit of NFκB was a common denominator for the two tumor groups.

**Table 1:** Transcription binding motif analysis of common DE genes and conditions between DE genes.

Common Gene Conditions	TFBMs						
Common DEs up-regulated among all samples (p<0.002, FDR<40%)	V$ELK1_01	V$NRF2_01	V$SRF_Q6	V$E2_Q6			
Common DEs down-regulated among all samples (p<0.01, FDR<37.5%)	V$ARP1_01	V$AHRARNT_01	V$AP2_Q6	V$VMAF_01	V$ISRE_01	V$SP1_01	V$CDXA_01
Common DEs among pT1-Grade II and pT1-Grade III (p<0.002, FDR<30%)	V$AP4_01	V$RORA2_01	V$EGR1_01	V$MZF1_01			
Common DEs among pT1-Grade II and pT2-pT3 Grade III (p<0.0002, FDR<10%)	V$AHRARNT_01	V$EVI1_05	V$OCT1_03	V$HEN1_02	V$CAP_01		
Common DEs among pT1-Grade III and pT2-pT3-Grade III (p<10^−5^, FDR<20%)	V$NFKB_C (p = 10^−10^)	V$NFKAPPAB65_01 (p = 10^−6^)	V$GRE_C	V$CREBP1_01	V$EVI1_03		
Common DEs up-regulated in pT1-Grade II and down-regulated in pT1-Grade III	**Non significant**						
Common DEs up-regulated in pT1-Grade II and down-regulated in pT2-pT3Grade III (p = 0.0002, FDR<40%)	V$VJUN_01						
Common DEs up-regulated in pT1-Grade III and down-regulated in pT2-pT3Grade III (p<0.0002, FDR<20%)	V$E2F_02	V$CREBP1_01	V$IK3_01				
Common DEs down-regulated in pT1-Grade II and up-regulated in pT1-Grade III (p<0.0005, FDR<20%)	V$YY1_02 (p = 10^−7^)	V$AP4_01	V$E2F_02	V$ELK1_01			
Common DEs down-regulated in pT1-Grade II and up-regulated in pT2-pT3Grade III	**Non significant**						
Common DEs down-regulated in pT1-Grade III and up-regulated in pT2-pT3Grade III (p<0.0002, FDR<40%)	V$EGR1_01	V$EGR2_01					

Attention is drawn to two very important transcription factors YY1 and NFκB. It appeared that down-regulated genes in T1-Grade II and up-regulated in T1-Grade III are commonly regulated by YY1. Also, common DE genes between T1-Grade III and T2- pT3-Grade III appear to have transcription factor NFκB as a common denominator.

#### Chromosome Mapping: CodeLink Platform

**Figure 9:**
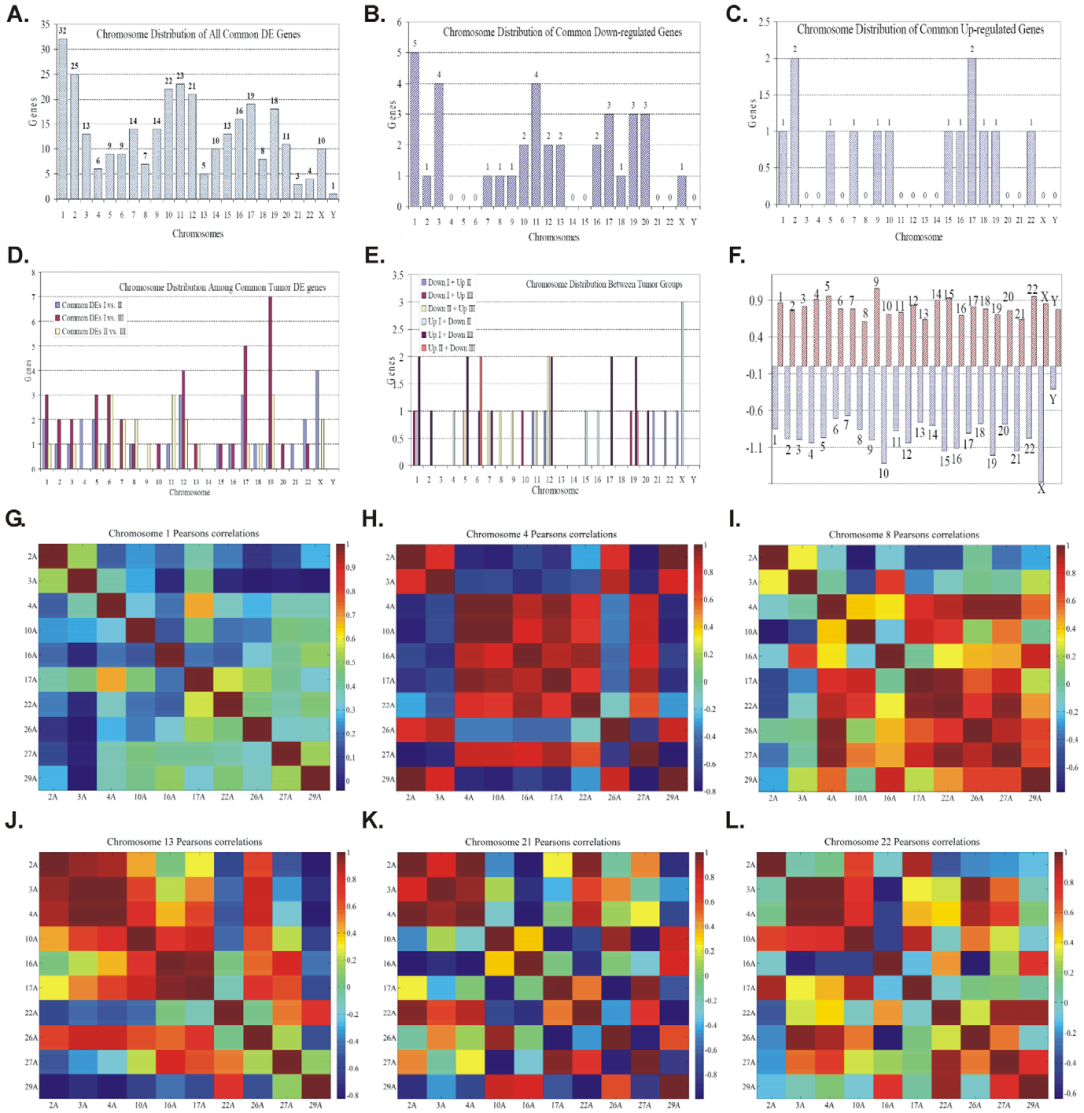
Chromosome distribution of common differentially expressed genes. Common genes between all samples (A) showed peaks of gene expression in chromosome 1, 11 and 19. Down-regulated genes (B) showed peaks of gene expression in chromosome 1 and 11. Up-regulated genes (C) showed a peak in chromosomes 1 and 7. In concordance gene expression manifested a peak in chromosome 19 for common DE genes between groups pT1-Grade II (group I) and pT2- pT3-Grade III (group III) (D), while chromosome X appeared to express most of genes between down-regulated genes in group pT1-Grade III (group II) and simultaneously in up-regulated genes in group pT2- pT3-Grade III (group III) (E). The median expression of all samples with respect to chromosomes is presented in (F) (numbers above and below bars indicate the chromosome). It appeared that the most active chromosome is chromosome 9 for all tumor samples while controls manifest maximum median activity at chromosome 10 and X. Correlation maps for all chromosomes has revealed some patterns within chromosomes 1 (G), 4 (H), 8 (I), 13 (J), 21 (K), 22 (L). Especially on chromosome 4 it appeared that there is both negative as well as positive co-expression for the majority of the tumor samples.

Chromosome distributions of the expressed data were also carried out in an attempt to search for more patterns in gene expression with respect to chromosome gene expression distribution. Chromosome distribution of the common DE genes is presented in **Figure 9**.

Common DE genes among all BC samples showed peaks of gene expression in chromosomes 1, 2 and 11 (**Figure 9A**). Chromosome 1 contained 32 (10.2%) DE genes, followed by chromosomes 2 and 11, which contained 25 (7.9%) and 23 (7.3%) DE genes, respectively. Chromosome 21 had the least number of DE genes (n = 3; 0.9%).The down-regulated genes exhibited peaks of gene expression in chromosomes 1 (5/36; 13.8%), 3 (4/36; 11.1%) and 11 (4/36; 11.1%). Chromosomes 4, 5, 6, 14, 15, 21, 22 and Y, did not present any down-regulated gene (**Figure 9B**). The up-regulated genes showed peaks in chromosomes 1 (2/14; 14.2%) and 7 (2/14; 14.2%); whereas chromosomes 1, 5, 7, 9, 10, 15, 16, 18, 19 and 22 contained 1 up-regulated gene (1/14; 7%) (**Figure 9C**). In concordance, gene distribution manifested a peak in chromosome 19 for common DE genes between groups T1-Grade II and T2/T3-Grade III (**Figure 9D**), while chromosome X appeared to express most genes between down-regulated genes in group T1-Grade III and simultaneously in up-regulated genes in group T2/T3-Grade III (**Figure 9E**).

Furthermore, we examined the expression of all genes as distributed on each chromosome. This was performed by evaluating the mean gene expression for up- and down-regulated genes separately, since up-regulated genes are those that are active in tumor samples as compared to control and down-regulated genes are those that are active in control samples as compared to tumor samples. Hence, for tumor samples the maximum activity occurred on chromosomes 9, 22, X and for control samples on chromosomes 10, 19 and X.

A chromosomal correlation analysis was also carried out, as previously described by *Cohen et al.* [30]. We used chromosomal correlation maps to reveal common expression patterns among genes (**Figure 9G–L**). In particular, we searched for common chromosomal expression among all chromosomes. Those manifesting certain correlation patterns included chromosomes 1 (**Figure 9G**), 4 (**Figure 9H**), 8 (**Figure 9I**), 13 (**Figure 9J**), 21 (**Figure 9K**) and chromosome 22 (**Figure 9L**), with chromosome 4 manifesting the most prevalent correlation pattern (**Figure 9H**). The fewest genes were mapped on chromosomes 13 and 21, which indicated that gene activity does not correlate with the number of genes active on a chromosome.

#### Functional Categories of differentially expressed genes and Gene Ontology Annotation: CodeLink Platform

**Figure 10:**
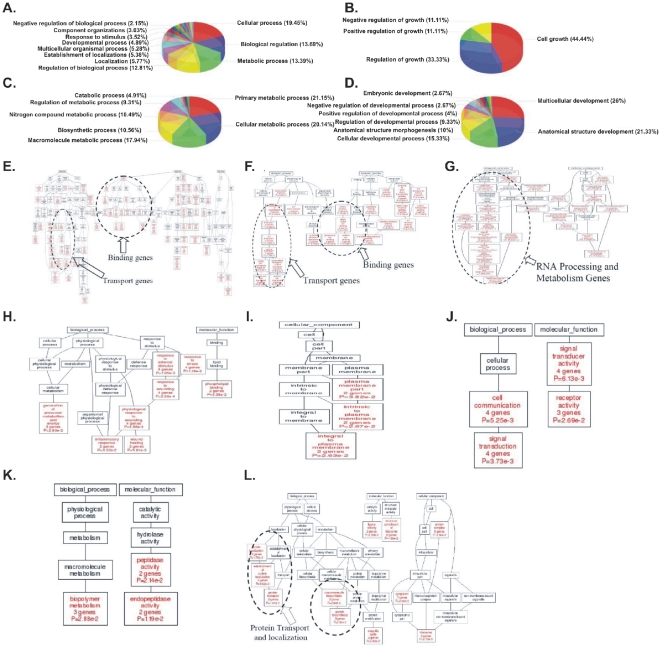
Thirty-one percent (30.94%) of genes were attributed to Biological Process. Within biological processes (A) genes for growth (B), metabolism (C) and development (D) were selected. Dendrograms (DAG trees) of Gene Ontology analysis of known differentially expressed genes in all combinations as they are presented in Table S5 was performed. Interestingly, common up-regulated genes were attributed to RNA processing and metabolism (G). The results include those combinations that have manifested significant function annotations at the *p*<0.05 level.

Gene definitions were used according to the NCBI (http://www.ncbi.nlm.nih.gov). Each gene can belong to more than one category. Categories of known genes were *Biological Process* (30.94%), *Cellular Component* (34.66%) and *Molecular Function* (33.76%). Furthermore, biological process was divided as presented in **Figure 10**. We searched further into the biological process category, and the results of the functional annotation are presented in **Figure 10B–D**. From the immense number of functions of the DE genes, five categories were outlined: cell death, cell growth, metabolism, development and RNA processing. Genes related to cell death included *MALT1*, *RHOT2*, *SON*, *CECR2*, *F2*, *PDE1B*, *PAK1*, *PLA2G6*, *CRADD*, *DNM1L*, *PDCD7*, *PUF60*, *ADAMTSL4*, *PERP*, *MARK4*, *DIDO1* and *BCL2L1*. Genes related to cell growth included *TFCP2L1*, *PRKCQ*, *FGF20*, *NDRG3* and *FHL1*. Genes related to embryonic development included among others *ADAM10*, *TNFRSF17*, *MYF5*, *CAP2* and *NDRG3*. Genes related to metabolism included among others *ATP2B4*, *GOLGA2L1*, *MMP24*, *RPLP1*, *APOC2*. Finally, among the RNA processing and transcription regulation genes were *ZNF132*, *ZNF135 MYF5*, *PPARG*, *ATOH8* and others.

Cell death-related genes had no other subcategories. Cell growth-related genes were further divided into negative and positive regulation of cell growth (11.1% respectively), regulation of growth (33.33%) and cell growth *per se* (44.4%). Metabolism-related genes were subdivided into catabolic processes (4.91%), regulation of metabolic processes (9.31%), nitrogen compound metabolic process (10.49%), biosynthetic processes (10.66%), macromolecule metabolic processes (17.94%), cellular metabolic processes (20.14%) and primary metabolic processes (21.15%). Finally, developmental genes were further divided into embryonic development (2.67%), negative regulation of embryonic development (2.67%), positive regulation of developmental processes (9.33%), regulation of developmental processes (9.33%), anatomical structure morphogenesis (10%), cellular developmental processes (5.33%), anatomical structure development (21.33%) and multicellular organismal development (26%).

In order to gain more insight into gene functions GO analysis was performed on the groups of commonly regulated genes as they were described in **Table 1**. Dendrograms were used with the hypergeometric test for determining statistical significance, as described by *Zhang et al.* [35], [37] for genes that were differentially expressed between tumor samples and tumor groups, respectively. **Figure 10E–L** documents the significant (p<0.05) gene annotations that were found with Gene Ontology analysis. Attention should be focused on two categories: In the common down-regulated genes, the prevalence of transport and binding genes was observed (**Figure 10E**). At the same time, the prevalence of RNA metabolism and processing genes among the up-regulated genes was significant (**Figure 10G**). Regarding the DE genes that were down-regulated in tumors of T1-Grade III and were simultaneously up-regulated in tumors of T2/T3-Grade III, genes were identified whose significant functions were cell-cell communication and signal transduction (**Figure 10J**).

### Comparison between two groups: all tumor samples versus all control samples

**Figure 11:**
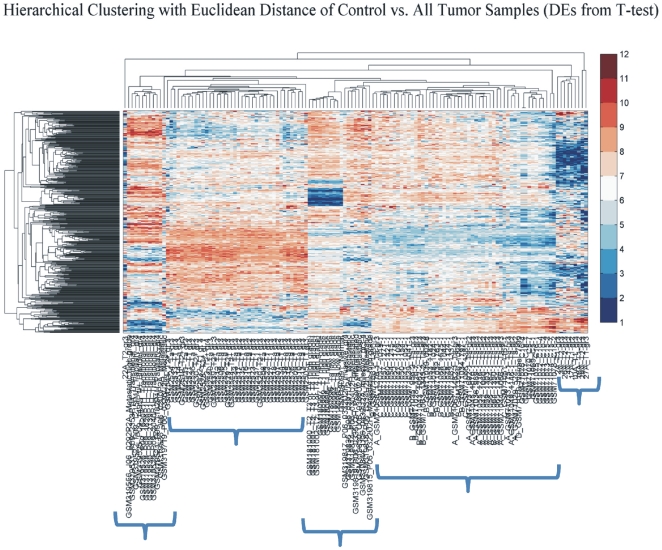
Hierarchical clustering (HCL) of between all control and all tumor samples, both considered as two separate groups. HCL made distinctions between the different platforms, indicating that the DE genes were adequate to do such a classification. Hence, similarities from this group would be expected to be due to the tissues *per se*.

**Figure 12:**
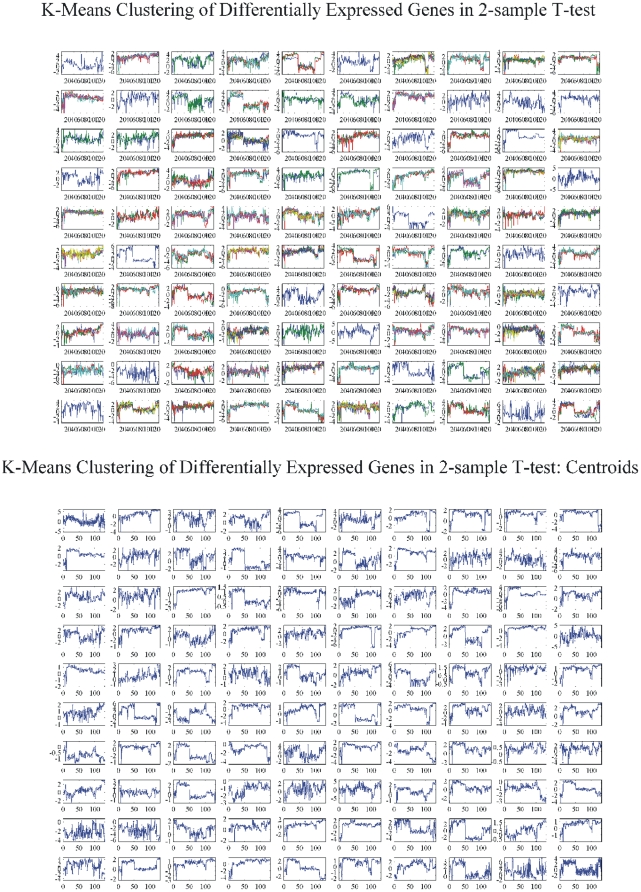
K-means clustering of DE genes between all tumor groups and all control samples.

A two-sample T-test was performed in order to identify DE genes between those two groups. This analysis revealed 434 DE genes. Hierarchical clustering (HCL) showed a clear distinction among tumor samples (**Figure 11**). It clustered groups of genes according to their tumor class. *k-means* clustering on this gene subset was also carried out in order to detect commonly expressed genes (**Figure 12**). From the cluster analysis we were able to identify three groups of genes that were down-regulated in all samples: Clusters 79, 81, 82 represent genes that were down-regulated in the majority of genes. Specifically, these genes were *BMP4*, *CRYGD*, *DBH*, *GJB1*, *KRT83*, *MPZ*, *NHLH1*, *TACR3* in cluster 79; *ACTC1*, *MFAP4*, *SPARCL1*, *TAGLN* in cluster 81; and *TPM2* in cluster 82. No gene was simultaneously up-regulated in all of the samples.

#### CDC20: A common marker among tumor groups

**Figure 13:**
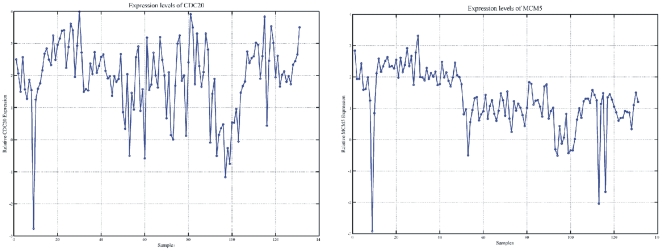
*CDC20* expression across all samples. Apart from 9 samples, the gene appeared to be up-regulated in the rest 120 samples.

Tumor samples were separated into groups as described in **Table S1**. Each group was compared against all control samples and identified DE genes. The goal of this analysis was to identify genes that were simultaneously DE in all tumor groups. Indeed, we identified one known gene, the cell division cycle 20 homolog (Gene ID: 991) or *CDC20*. *CDC20* is a cell cycle regulator among other functions, and, to date, there are no other reports linking it to bladder cancer. *CDC20* appeared to be commonly differentially expressed in all tumor groups, except for the Ta-grade3 group. Its expression levels are depicted in **Figure 13**. *CDC20* appeared to be over-expressed in the majority of the tumor samples.

#### LHCGR is the most common DE gene among the individual tumor samples: The Intra-experimental Case

**Figure 14:**
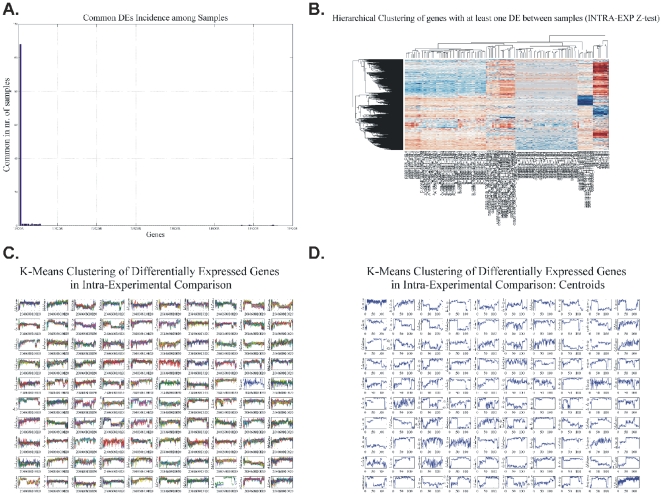
*LHCGR* expression (A) and HCL of differentially expressed genes analyzed with one-sample z-test (B). (C) K-means clustering of DE genes in Intra-experimental comparison. (D) centroids.

We compared genes in tumor samples individually, as ratios, within the same experiment. Our analysis showed that *LHCGR* was the most common DE gene found among the tumor samples. In particular, *LHCGR* was differentially expressed in 108/129 (83.7%) samples (**Figure 14A**). We also outlined the genes that appeared to be differentially expressed in at least one BC sample. In this group both HCL and k-means clustering were performed (**Figure 14**). However, no further groups of genes with common expression were revealed.

#### Common genes that were not differentially expressed: The Inter-experimental case

**Figure 15:**
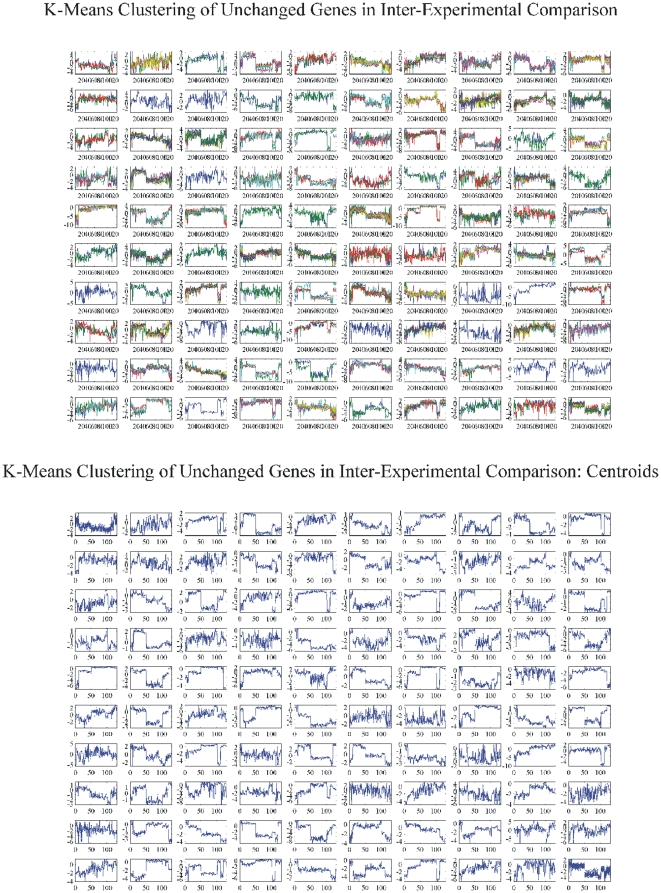
K-means clustering of unchanged genes in the inter-experimental comparison.

Similarly, as in the case of Intra-experimental comparisons, we searched for DE genes among all tumor samples. However, no surprising results were obtained since DE genes were expected to be encountered across such a wide range of samples. Yet another group of genes triggered our interest: those genes that were not DE across all tumor samples. Their importance lies in the fact that they were similar in all bladder cancer types, regardless of population, sampling method or microarray platform and experimentation procedure. The results are presented in Figure 15. However, they did not give groups of similar expression.

#### Combining the two cases: Intra-experimental vs. Inter-experimental

**Figure 16:**
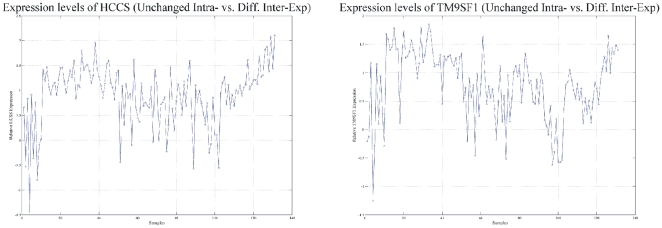
*HCCS* and *TM9SF1* were simultaneously unchanged in the intra-experimental and differentially expressed in the inter-experimental comparisons. Expression profiles of *HCCS* (A) and *TM9SF1* (B).

Since the analysis of individual samples did not provide distinct gene groups, we searched for genes that were: (a) unchanged in the intra-experimental, and DE in the inter-experimental comparisons; and (b) unchanged in the inter-experimental and DE in the intra-experimental comparisons. For case (a), two genes fulfilled both requirements: *HCCS* (holocytochrome c synthase; ID: 3052) and *TM9SF1* (transmembrane 9 superfamily member 1; ID: 10548) (**Figure 16**). Moreover, the comparison for case (b) revealed *LHCGR* (luteinizing hormone/choriogonadotropin receptor; ID: 3973), a gene that also appeared to be the most common DE gene among the tumor samples.

#### Transcription Factor Binding Motif (TFBM) Analysis: Cross-Platform Comparisons

**Figure 17:**
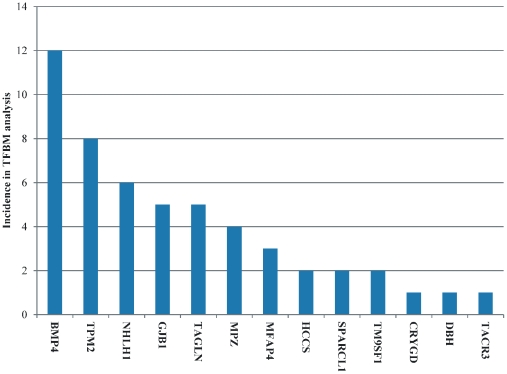
Incidence of genes among predicted transcription factors. The most prevalent gene was *BMP4*.

Across the commonly expressed genes that were identified in the majority of tumor samples, TFBM analysis was performed in order to identify transcription factors (TFs) which might affect expression of the genes. The TFs predicted by our analysis are summarized in Table 2. Unexpectedly, the glucocorticoid receptor (GR) was predicted as one of the TFs in the common gene set. In order to find which gene was most commonly represented among the TFs, we plotted the incidence of each gene as a function of the times of appearance within the predicted TFs (Figure 17). The gene BMP4 (bone morphogenetic protein 4; ID: 652) had the most binding sites for the predicted TFs. To our surprise, *CDC20* and *LHCGR* were not represented in the TFBM analysis.

**Table 2:** Predicted transcription factors (TFs) for 14 out of 17 genes commonly regulated in bladder cancer samples.

TFBM	Inc.	Gene IDs and Names							
_WTGAAAT_UNKNOWN	4	8404	1421	4807	6870	SPARCL1	CRYGD	NHLH1	TACR3
_CCAWWNAAGG_V$SRF_Q4	2	7169	6876			TPM2	TAGLN		
_V$STAT3_02	2	652	10548			BMP4	TM9SF1		
_RNGTGGGC_UNKNOWN	3	2705	652	4359		GJB1	BMP4	MPZ	
_V$HMEF2_Q6	2	652	7169			BMP4	TPM2		
_V$SRF_Q4	2	7169	6876			TPM2	TAGLN		
_V$SMAD_Q6	2	652	6876			BMP4	TAGLN		
_V$HEN1_01	2	652	4239			BMP4	MFAP4		
_V$SRF_Q6	2	7169	6876			TPM2	TAGLN		
_TTGTTT_V$FOXO4_01	4	8404	652	3052	4359	SPARCL1	BMP4	HCCS	MPZ
_V$ZIC3_01	2	2705	4807			GJB1	NHLH1		
_V$TTF1_Q6	2	652	7169			BMP4	TPM2		
_V$GR_Q6_01	2	4359	4807			MPZ	NHLH1		
_V$HEN1_02	2	652	4239			BMP4	MFAP4		
_V$ZIC1_01	2	2705	7169			GJB1	TPM2		
_V$SRF_Q5_01	2	7169	6876			TPM2	TAGLN		
_V$CEBPB_02	2	1621	4807			DBH	NHLH1		
_V$POU1F1_Q6	2	652	4807			BMP4	NHLH1		
_SCGGAAGY_V$ELK1_02	3	2705	10548	3052		GJB1	TM9SF1	HCCS	
_CAGGTG_V$E12_Q6	4	2705	652	4239	4807	GJB1	BMP4	MFAP4	NHLH1
_YNGTTNNNATT_UNKNOWN	2	652	7169			BMP4	TPM2		
_WGGAATGY_V$TEF1_Q6	2	652	4359			BMP4	MPZ		

#### Chromosome Mapping: Cross-Platform Comparisons

**Figure 18:**
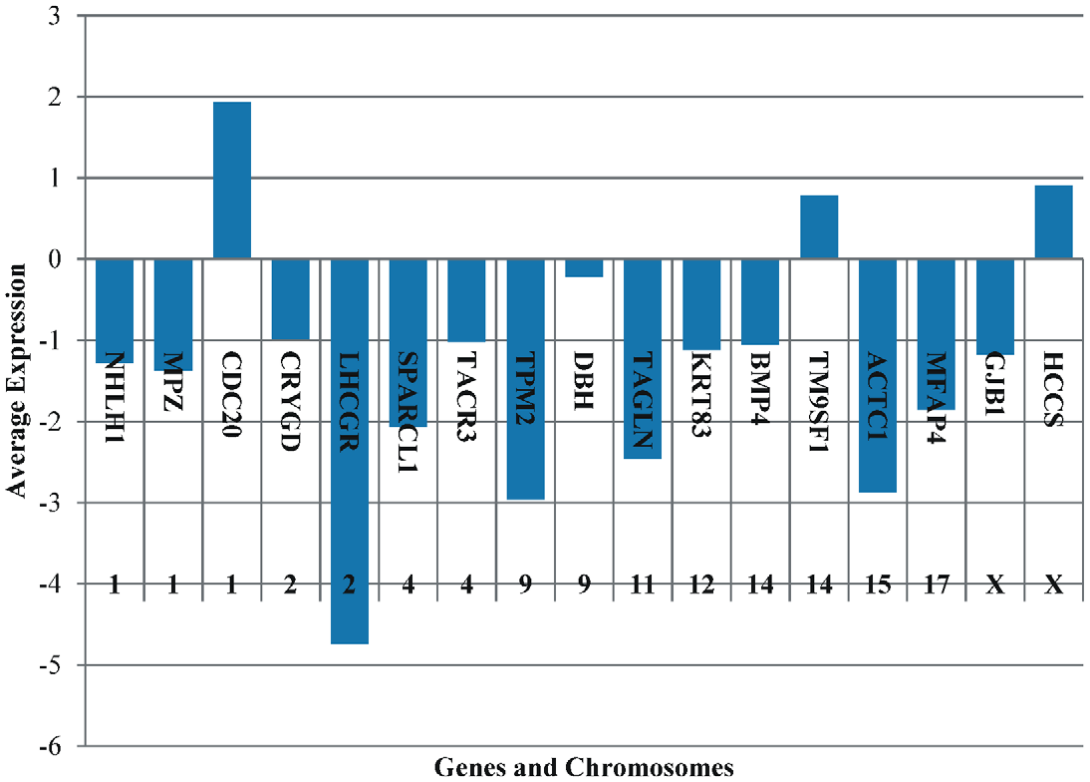
Average gene expression with respect to their corresponding chromosomes.

Chromosome mapping was performed with the genes that were identified as common, among all BC samples (**Figure 18**). In BC, most chromosomes had inactivated (down-regulated) genes, versus the control samples. However, two genes were an exception: *CDC20* (in chromosome 1) and *HCCS* (in chromosome X).

#### Pathway analysis of common genes

Considering that the genes which were identified as being common play an important role in bladder cancer, we further attempted to isolate those genes that may play a role in tumoral “cross-roads”. In order to do so, we hypothesized that the genes which would fulfill such a requirement should participate in more than one familiar pathways. Indeed, we encountered four genes that participated in eight different pathways, as depicted in **Table 3**.

**Table 3:** Pathway participation of common genes in bladder cancer.

	Pathway	Genes					Average expression	
**KEGG**	Cardiac muscle contraction	70	7169	ACTC1	TPM2	p<0.01	**−2.8730277**	**−2.9625465**
	Dilated cardiomyopathy	70	7169	ACTC1	TPM2	p<0.01	**−2.8730277**	**−2.9625465**
	Hypertrophic cardiomyopathy (HCM)	70	7169	ACTC1	TPM2	p<0.01	**−2.8730277**	**−2.9625465**
	Calcium signaling pathway	3973	6870	LHCGR	TACR3	p<0.01	**−4.7375153**	**−1.0159812**
	Neuroactive ligand-receptor interaction	3973	6870	LHCGR	TACR3	p<0.01	**−4.7375153**	**−1.0159812**
**Pathway Commons**	Signaling by GPCR	3973	6870	LHCGR	TACR3	p<0.01	**−4.7375153**	**−1.0159812**
	Class A/1 (Rhodopsin-like receptors)	3973	6870	LHCGR	TACR3	p<0.01	**−4.7375153**	**−1.0159812**
**Wikipathways**	Striated Muscle Contraction	70	7169	ACTC1	TPM2	p<0.01	**−2.8730277**	**−2.9625465**
	Peptide GPCRs	3973	6870	LHCGR	TACR3	p<0.01	**−4.7375153**	**−1.0159812**

Four genes (*ACTC1*, *TPM2*, *LHCGR*, *TACR3*) appeared to participate in 8 different pathways.

#### Functional Categories of differentially expressed genes and Gene Ontology Annotation: Cross-Platform Comparisons

**Figure 19:**
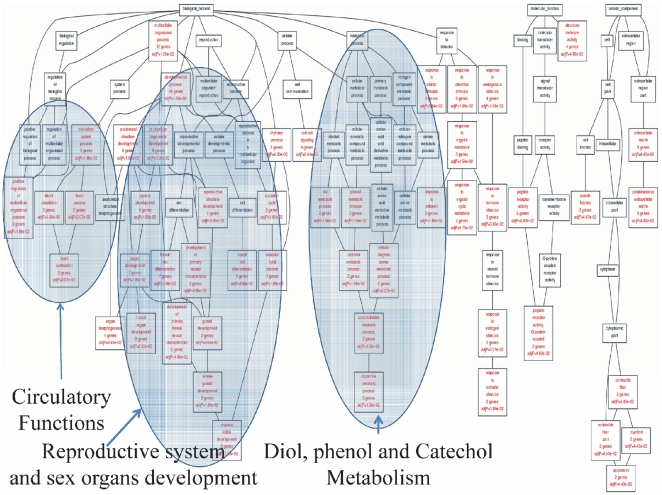
GO terms annotation of the common gene set. Three functions could be outlined a) circulatory functions, b) reproductive organ development and catecholamine metabolism.

Finally, GO terms in which the genes participated were analyzed (**Figure 19**). Three main functions were outlined: a) circulatory system regulation, b) reproductive organ and sex development, and c) catecholamine metabolism. This enrichment showed that the predicted gene set has more than a dual role.

## Discussion

Several studies have focused on the expression profiling of urinary bladder cancer using microarrays. The purpose of these studies was the classification of bladder cancer, the definition of biological phenotypes, the identification of gene expression patterns in superficial and invasive human bladder cancer, the identification of superficial, muscle-invasive, and metastasizing transitional cell carcinoma of the bladder. [17], [18], [38], [39], [40], [41], [42], [43], [44], [45]. In the present study, the experimental pathologic cases were carefully selected in order to obtain at least one pair from each tumor group: T1-Grade II, T1-Grade III and T2/T3-Grade III. Furthermore, in our pooled microarray analysis, a wide range of data from publicly available microarray datasets was included, increasing the number of specimens under investigation, to 129 BC samples and 17 controls in total.

To date, the main approach in microarray analysis has been the discovery of differences between pathogenic samples. The purpose of these studies was to detect patterns that discriminate between further subcategories of pathogenic samples, such as tumors. However, the fact that, instead of bearing differences, various tumors can also possess many similarities explained by their common origin or by a common general mechanism of tumorigenesis, has been neglected. Since tumors do possess similar characteristics regardless of the tumor type, it would be reasonable to assume that such common phenotypical manifestations would be reflected from genomic i.e. transcriptional similarities. Therefore, we focused our analysis, not only on the differences between tumor samples, but also on their similarities. We focused on the genes that simultaneously exhibited differential expression (either up- or down-regulation) among the different subclasses of BC, compared to the normal tissue.

Therefore, instead of searching for classification patterns between tumor samples based on gene expression profiles we used a reverse engineering approach in order to search for common patterns among different tumor samples. Since there are expected differences between even similar samples, simply due to the diversity that characterize biological systems, the chances of finding common mechanisms are scarce. However, one could argue that even if similarities are found, these may be attributed to the fact that, from a huge gene pool, several similarly expressed genes are expected to exist. Yet, if this process is random, then similarities should include genes or functions that do not provide any meaning with regards to the tumor/samples under study. In other words, if we detect common genes with similar expression among different, unconnected samples, this implies that several common mechanisms among tumors, should indeed exist. In order to include population and platform bias, we used all the available control samples from all the microarray platforms that we studied. *Goldstein et al.* and *Hirsch et al.* [9], [46], investigated common signatures for BC and cancer/lipid metabolism. These studies suggest that biological systems on their way to disease follow similar paths. Similarly, our approach aimed to identify similar patterns among groups of different BC types.

### Clustering and classification

#### The CodeLink Case

We initially determined gene groups among the BC samples. This approach outlined differences between samples. We identified 831 genes that were differentially expressed in all 10 tumor samples simultaneously. On the other hand, k-means clustering outlined several groups with differential expression, such as clusters 3, 4, 5, 10, 12, 14 in **Figure 3B**. When clustering for DE genes using only the k-means algorithm, no clear distinction were detected among individual samples, meaning that there were no clear differences among samples. Furthermore, we investigated the presence of common profiles, searching for simultaneously common up- and down-regulated genes, among all tumor samples. Thirty-three genes were up-regulated and 85 genes were down-regulated in all 10 BC samples vs. the 5 normal tissues, simultaneously. The first finding from classification analysis was that normal urothelium, superficial, and muscle-invasive bladder cancers, showed distinct gene expression profiles, as revealed by hierarchical clustering and principal component analyses (**Figure 8E**). Yet, PCA analysis indicated two main groups of expression profiles in all samples when tested individually and not in groups (**Figure 6E**). On the other hand, when PCA analysis was carried out for the DE genes, only the samples were grouped into additional clusters (**Figure 7E–G**). K-means clustering of all genes and all samples, as well as clustering of tumor groups, revealed 49 clusters. Moreover, k-means clustering of common DE genes in all samples revealed 24 clusters.

We did not identify any genes that were commonly DE in all tumor groups, simultaneously. Yet, we identified such genes in tumor group pairs. Thirty-eight common DE genes were identified between tumor groups T1-Grade II and T1-Grade III; 44 DE genes were identified between groups T1-Grade II and T2/T3-Grade III; and 33 DE genes were identified between groups T1-Grade III and T2/T3-Grade III. The DE genes were also analyzed in 6 different combinations between paired tumor groups, i.e. all possible combinations of DE genes that were up-regulated in one tumor group, and down-regulated in the other (**Table S5**).

#### Cross-Platform Comparisons

The cross-platform analysis after the inclusion of a larger microarray dataset, provided us with an additional view of our concept. The number of common DE genes was now confined to a smaller group of genes. In total, 17 genes appeared to be commonly expressed among all BC samples: *BMP4*, *CRYGD*, *DBH*, *GJB1*, *KRT83*, *MPZ*, *NHLH1*, *TACR3*, *ACTC1*, *MFAP4*, *SPARCL1*, *TAGLN*, *TPM2*, *CDC20*, *LHCGR*, *TM9SF1* and *HCCS*.

In regards to *BMP4*, there is only one previous report linking it to bladder cancer, which suggests that its expression plays a growth inhibitory role [47]. To date, there is no publication indicating a connection among *CRYGD*, *GJB1*, *KRT83*, *MPZ*, *NHLH1*, *TACR3*, *ACTC1*, *MFAP4*, *SPARCL1*, *TAGLN*, *TPM2*, *LHCGR*, *TM9SF1* and bladder cancer. Future investigation needs to confirm the implication of these genes in urinary bladder cancer. Cluster analysis revealed a common group of genes when all controls and all BC samples were compared as two separate groups. For the majority of the genes, there are no known reports linking them to bladder cancer. Notably, regarding the commonly regulated *CDC20*, Kidokoro et al. recently mentioned that it has potential therapeutic properties [48].

### TFBMs

#### The CodeLink Case

Regarding TFBM analysis, Yin Yang 1 (YY1) and NFκB were among the most common transcription factors regulating the expression of the identified DE genes. The transcription factor (TF) YY1 commonly regulated the expression of the down-regulated genes in T1-Grade II and simultaneously in T1-Grade III. YY1 has been identified to target a plethora of potential target genes, the products of which are important for proliferation and differentiation, and has therefore been proposed as an important prognostic marker for several human tumors [49], [50], [51], [52], [53]. The mechanisms of YY1 action are related to its ability to initiate, activate, or repress transcription depending on the context in which it binds. YY1 over-expression has been reported to affect the clinical behavior of several cancer types [54], [55]. Recently, a dual role of YY1 in cancer development has been suggested, either through over- or under-expression, depending on the tumor type. In bladder cancer, significant differences have been detected between superficial TCC with carcinoma *in situ*, and normal specimens, as well as between muscle-invasive carcinoma and normal tissue [56].

NFκB, on the other hand, appeared to regulate common DE genes between tumors of T1-Grade III and those of T2/T3-Grade III. In particular, the p65 subunit of NFκB was a common denominator for the two tumor groups. *Zhong et al.* demonstrated that transcriptionally inactive nuclear NFκB in resting cells consists of homodimers of either p65 or p50 complexed with the histone deacetylase HDAC1 [57]. Only p50-HDAC1 complexes bound to DNA and suppressed NFκB-dependent gene expression in unstimulated cells. Appropriate stimulation caused nuclear localization of NFκB complexes containing phosphorylated p65 that associated with CBP and displaced the p50-HDAC1 complexes. These results demonstrated that phosphorylation of p65 determines whether it associates with either CBP or HDAC1, ensuring that only p65 entering the nucleus from cytoplasmic NFκB -IKB complexes can activate transcription. The inhibitory protein, NFKBIA, sequesters the transcription factor, NFκB, as an inactive complex in the cytoplasm.

#### Cross-Platform Comparisons

BMP4 appeared to be the gene with the most predicted transcription factor binding sites. Also, when we examined the CodeLink platforms individually, NF-κB was identified as a TF with a putative role in gene regulation. Moreover, in the cross-platform comparisons we identified the GR as a significantly implicated transcription factor. Interestingly, both GR and NF-κB are two TFs that are in interplay, at least in inflammation. The role of GR is already known in hematologic malignancies. The role of NF-κB has also been mentioned in drug resistance of neoplasias. However, their role in BC is still unknown. Their appearance in the present analysis, indicates a putative implication. GR has previously been mentioned to participate in the oncogenesis of bladder cancer [58]. Yet, it is still unclear whether the GR plays a role in BC. Another TF predicted by our analysis was STAT3, previously mentioned to be expressed in BC initiating cells [59].

### Chromosome Mapping

#### The CodeLink Case

**Figure 20:**
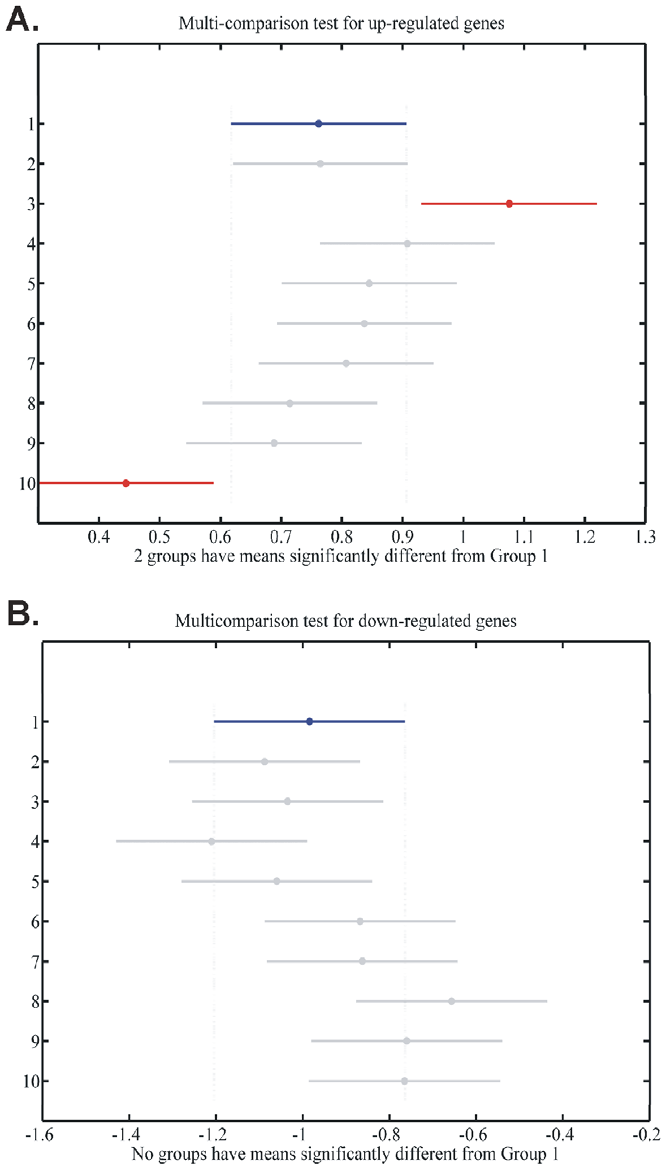
One-way ANOVA showed significant differences between samples 4A and 29A in up-regulated genes (A) while there were no significant differences in down-regulated genes (B) as both groups were mapped on chromosomes.

Chromosome mapping may be proven to be a useful tool in the detection of gene expression patterns. One-way ANOVA test showed significant differences between samples 4A and 29A in up-regulated genes, as far as chromosome gene expression is concerned (*p<0.05*) (**Figure 20A**), while no differences were observed between down-regulated genes (**Figure 20B**). The mean gene expression showed a maximum on chromosome 9 (**Figure 9F**). This was interesting since the relations between chromosome 9 and BC have been previously reported [60], [61]. Chromosome 9 has been reported to undergo deletion of its long arm. Although, chromosome 1 manifested a peak in gene number distribution (**Figure 9A**), gene expression did not correlate with genes. Moreover, on chromosome 9, three genes belonged to the commonly expressed genes. These were *LRRC8A* (commonly down-regulated), *C9orf103* and *PTPDC1* (commonly up-regulated). There are no reports for these three genes in regards to their relationship to BC. Our attention was drawn to chromosome X, which is generally known to possess active genes in cancer cells that are silenced in somatic cells. In chromosome X, the gene FHL1 belonged to the commonly down-regulated genes. Notably, it was recently reported that this gene is hypermethylated and contributes to the invasion and migration of BC [62]. Its expression among all samples, irrespective of the tumor type, makes it an attractive target for further investigation as a marker for tumor cell migration and invasion.

In order to gain further insight on gene expression with respect to correlations between BC samples, we constructed correlation maps as previously reported [30]. We searched for patterns among all chromosomes. Yet, the most interesting pattern was manifested in chromosome 4 which showed the greatest number of positive and negative regulation between samples (**Figure 9H**). In chromosome 4, six genes were mapped: *TACR3*, *RNF150*, *ANXA10*, *CENTD1*, *EXOC1* and *GRSF1*. Interestingly, *GRSF1* is a gene that is involved in RNA binding and also stimulation of translation of viral mRNAs *in vitro*. This gene was over-expressed in all samples except for case 29A, which is probably explained by the fact that all patients received BCG treatment. From these genes, none belonged to a common gene expression group, which makes the finding of correlated expression stronger. In particular, samples 4A, 10A, 16A and 17A showed positive correlation while 2A and 3A manifested negative regulation with samples 4A, 10A, 16A, 17A and 27A.

The gene TACR3 belongs to a family of genes that function as receptors for tachykinins. There are no reports on the role of *TACR3* in BC. Also, RNF150 a ring finger protein, has not been previously reported regarding its relationship to BC. Similarly, *ANXA10*, a gene that encodes a member of the annexin family is reported for the first time to be correlated with bladder cancer. Members of this family play a role in cell growth and signal transduction. Likewise, the gene CENTD1 which contains a RAS-associating homology domain has no previous reports regarding its relationship to BC.

Furthermore, in chromosome 8 (**Figure 9I**) the gene DENND3 was commonly down-regulated among the BC samples. There is no evidence linking it to BC. In chromosome 13 (**Figure 9J**), two genes were commonly down-regulated in the BC samples: *RXFP2* and *KL*. Notably, *RXFP2* has been reported to participate in male reproductive system malignancies and diseases. In a recent report, it was suggested that this gene is involved in uterine fibroids, where it was reported to be down-regulated in all diseased samples [63]. *RXFP2* is also considered to be a stimulator of genes promoting proteolysis such as the MMP and TIMP families [64].

Finally, the chromosomes with the majority of commonly up- or down-regulated DE genes were chromosomes 1 (6 genes), followed by chromosome 17 (5 genes). In chromosome 1, *OTUD7B* is a commonly down-regulated gene which has been reported to interact and regulate NFκB activity [65], [66], [67]. NFκB, was predicted from our analysis as a key factor regulating DE genes among Grade III tumors. The gene ADAMTSL4, commonly down-regulated, belongs to the ADAMTS family of proteins with cell adhesion and angiogenetic properties. It has been reportedly involved in adult acute leukemia and ovarian cancer, which makes its involvement in BC a significant finding [68], [69]. The gene ATF3, down-regulated in all samples, encodes for a transcription factor, and has been previously reported to be involved in BC, and in particular to be up-regulated in hTERT transformed cells [70]. *ACBD3*, commonly up-regulated, is a gene that participates in the maintenance of the Golgi structures of cells. It has been reported recently that the protein ACBD3 is released in asymmetric cell division in neural cells [71]. *RAB3B*, commonly down-regulated, participates in the regulation of exocytosis. It has been reported to play a role in rituitary adenomas [72], [73]. No reports have been found for the GPR153 gene.

In chromosome 17, the following genes belonged to the common expression groups: *NCOR1*, *GFAP*, *QRICH2*, *ANAPC11* and *PER1*. *NCOR1*, commonly up-regulated, is a transcription repressor of thyroid-hormone receptors. It has been reported to be linked to bladder cancer cells. In particular, it has been shown that over-expression of this gene was linked to the regulation of nuclear receptors such as PPARgamma and VDR, where PPARgamma (*PPARG*) was differentially expressed in our dataset. Interestingly, it has been reported that *NCOR1* over-expression provides a target for therapies with histone deacetylase inhibitors, such as vorinostat [74]. There are no reports for the role of *GFAP* in BC. *QRICH2* (glutathione rich 2) is a gene whose functions are unknown. Its appearance in BC makes an interesting target for further investigation. *ANAPC11*, commonly up-regulated, appears to be a very important factor in the regulation of cell cycle progression, whereas its aberrant expression is linked to tumor progression [75], [76]. The presence of this gene in our samples implies that it also plays a role in bladder cancer and cell cycle progression. It could also imply that its over-expression is a marker of good prognosis since all patients responded positively to therapy. *PER1*, commonly down-regulated, is a very important gene since it is the primary circadian pacemaker of the brain. Furthermore, it has been reported to suppress tumor cell proliferation [77] and its expression varies with aggressiveness due to polymorphisms of the gene in prostate cancers [78].

#### Cross-Platform Comparisons

In the case of cross-platform comparisons, inactivated genes were predicted. Also, three genes appeared to be activated i.e. over-expressed: CDC20, TM9SF1 and HCCS. Special interest was given to *HCCS*. It is located on the X chromosome and to date, there are no reports linking it to bladder cancer. Yet, it is one of the few activated genes that was common to all samples. *HCCS* is a mitochondrial gene, which means that it is inherited from the mother alone. Considering that mitochondrial dysfunction is closely related to cancer progression [79], [80], *HCCS* may play an interesting role in bladder cancer.

### Pathway Analysis

#### The CodeLink Case

In search for common pathways, when the CodeLink dataset was analyzed alone, the *urinary bladder cancer pathway* was identified as the first and most prevalent one.

#### Cross-Platform Comparisons

Since the Cross-Platform Comparisons identified 17 common genes among all of the tumor samples, the following question arose: how can we identify the most significant among them? Considering the multi-dimensional nature of cancer biology, there are more than one factor or gene that affects tumor behavior. This phenomenon is encountered in many aspects of cancer biology, from tumorigenesis to drug resistance and prognosis. Therefore, we hypothesized that the most significant genes, if any, should participate simultaneously in a variety of functions/pathways. The key aspect here is that all of them are common to all of the samples studied in the present work. Therefore, common pathway participation should also be expected to exist among samples. Hence, mapping those genes on known pathways revealed four genes that participate in 8 different pathways. The following were the most prevalent genes in pathway participation, and were found to be down-regulated in BC: *ACTC1*, *TPM2*, *LHCGR* and *TACR3*, To date, there are no reports linking them to BC. However, their inactivation in a variety of known pathways implies that they have a putative role. Interestingly, *LHCGR*, *ACTC1* and *TPM2* comprise the top-3 down-regulated genes compared to the control samples.

### GO analysis

#### The CodeLink Case

GO analysis and functional gene annotations provided further insight into the expression profile of the common genes. We searched within five main categories of GO annotations: cell death, cell growth, metabolism, development and RNA processing (**Figure 10**). First of all, we tested the genes for their functions and searched among those genes for the ones that manifested common expression patterns. Second, we analyzed common families of genes between the samples and tumor groups for significant functions.

Three cell death-related genes were commonly regulated in all samples: *PUF60*, *ADAMTSL4* and *BCL2L1*. *PUF60* was recently reported as a novel factor of tumor progression [81]. This is in agreement with the present study, since it was also up-regulated in the BC samples. *ADAMTSL4* was described in the previous section. Finally, *BCL2L1*, commonly down-regulated, is a mitochondrial gene expressed by the nucleus. It is localized in the mitochondrial membrane and facilitates the release of cytochrome C which is considered to be an effector of apoptosis. Consistent down-regulation of the BCL2L1 gene indicates that it could be considered for use as a prognostic or therapeutic marker in BC. The second category included developmental genes, i.e. genes known to participate in embryonic development (**Figure 10D**). *TNFRSF17* belongs to the Tumor Necrosis Family receptors. One of its functions is the activation of NFκB factor and it also participates in B-cell maturation. Also, it participates in embryonic B-cell development [82]. KL (*klotho*) encodes a type-I membrane protein that is related to beta-glucosidases. In a recent report, KL promoted apoptosis and growth inhibition in lung cancer cells [83]. This implies a similar function for BC. Regarding the metabolism-related genes we outlined: *NPC1L1*, *NCOA5* and *ELOVL3*. *NPC1L1* codes for a protein that takes up free cholesterol into cells through vesicular endocytosis and also participates in lipid metabolism. It has been indirectly linked to carcinogenesis through inhibition of its function [84]. *NCOA5* is a nuclear estrogen co-activator and hence indirectly linked to metabolism. It has been reported to regulate c-MYC expression as a downstream target of *TIP30* [85]. Finally, *ELOVVL3* participates in fatty acid chain elongation and formation of neutral lipids. It has been reported that this gene is controlled by steroid hormones in mouse models [86]. Lipid metabolism is involved indirectly with BC and in particular through the PPARG gene [87]. As an**other** example we could refer to the metabolism of arachidonic acid as an important tumor promotion factor [88]. The link between lipid metabolism and BC warrants further investigation.

Finally, analysis of all DE genes as well as the groups with common expression revealed the prevalence of transport and binding genes, and RNA processing genes (**Figure 10E–G**). In particular, prevalence of transport and binding genes was noted in the common down-regulated DE genes; the prevalence of RNA metabolism and processing genes in the up-regulated DE genes; as well as the prevalence of genes responsible for cell communication and signal transduction in the DE genes that were down-regulated in T1-Grade III tumors and up-regulated in T2/T3-Grade III tumors. The RNA processing genes included *NCOR1* (as previously discussed), *ZNF135* and *ATF3*. *ZNF135* is a zinc finger protein for which not much is known. It was consistently down-regulated in all samples, which denotes a possible role in BC.

#### Cross-Platform Comparisons

The GO analysis results were different in the case of *Cross-Platform Comparisons*. A total of 17 genes with several functions, apart from cell growth or cell death, was obtained. Thus far, there are no reports connecting catechol, diol, phenol or catecholamine metabolism with bladder cancer. Moreover, it was interesting to attribute developmental functions to the identified common genes.

### Conclusions

In the present work we employed microarray analyses in order to identify the common gene expression profile in bladder cancer. Previous gene expression studies have focused on identifying differences between tumor samples of the same type. Using a reverse engineering approach, we searched for common expression profiles among tumor samples. Through this investigation we were able to identify several important factors that warrant further investigation both as prognostic markers and as therapeutic targets. Such approaches may provide a better insight into tumorigenesis and tumor progression. The present findings reveal that tumors probably possess common characteristics. This type of gene expression analysis will provide further insights in the identification of universal tumor markers and will therefore aid in the development of more effective therapeutic approaches.

## Supporting Information

Figure S1 **The reference-design was the experimental design of the present microarray experiments (A).** Normal distributions of raw and normalized data (B). Box-plots of normalized log_2_-transformed ratios of samples against the average of controls (C).
(TIF)

Figure S2 **Results of cross-normalization between microarray samples.** All samples were normalized each with the respective recommended platform normalization method. A further normalization followed using quantile normalization algorithm.
(TIF)

Figure S3 ***p*** **-value Distribution of DE genes among tumor groups (A–C), along with FDRs (D–F).** Genes that obtained a *p*-value<0.05 were considered as DEs.
(TIF)

Figure S4 **Box-plots of tumor groups (A) and individual samples (B).** All samples in A and B with the exception of control averages are the log_2_ transformed ratios of samples over the control averages.
(TIF)

Table S1 **The categorization of BC samples into groups is presented.**
(TIF)

Table S2 **Common Differentially Expressed genes between Groups pT1-Grade II (Group I) and pT1-Grade III (Group II) (p<0.05).**
(DOC)

Table S3 **Common Differentially Expressed Genes between groups pT1-Grade II (Group I) and pT2- pT3-Grade III (Group III) (p<0.05).**
(DOC)

Table S4 **Common Differentially Expressed Genes between groups pT1-Grade III (Group II) and pT2- pT3-Grade III (Group III) (p<0.05).**
(DOC)

Table S5 **Groups of common genes in several combinations.**
(DOC)
