# Supplementary figures and images for: Identification of Common Differentially Expressed Genes in Urinary Bladder Cancer
Source: PLoS One. 2011 Apr 4;6(4):e18135. doi: 10.1371/journal.pone.0018135 (PMC3070717; doi:10.1371/journal.pone.0018135)

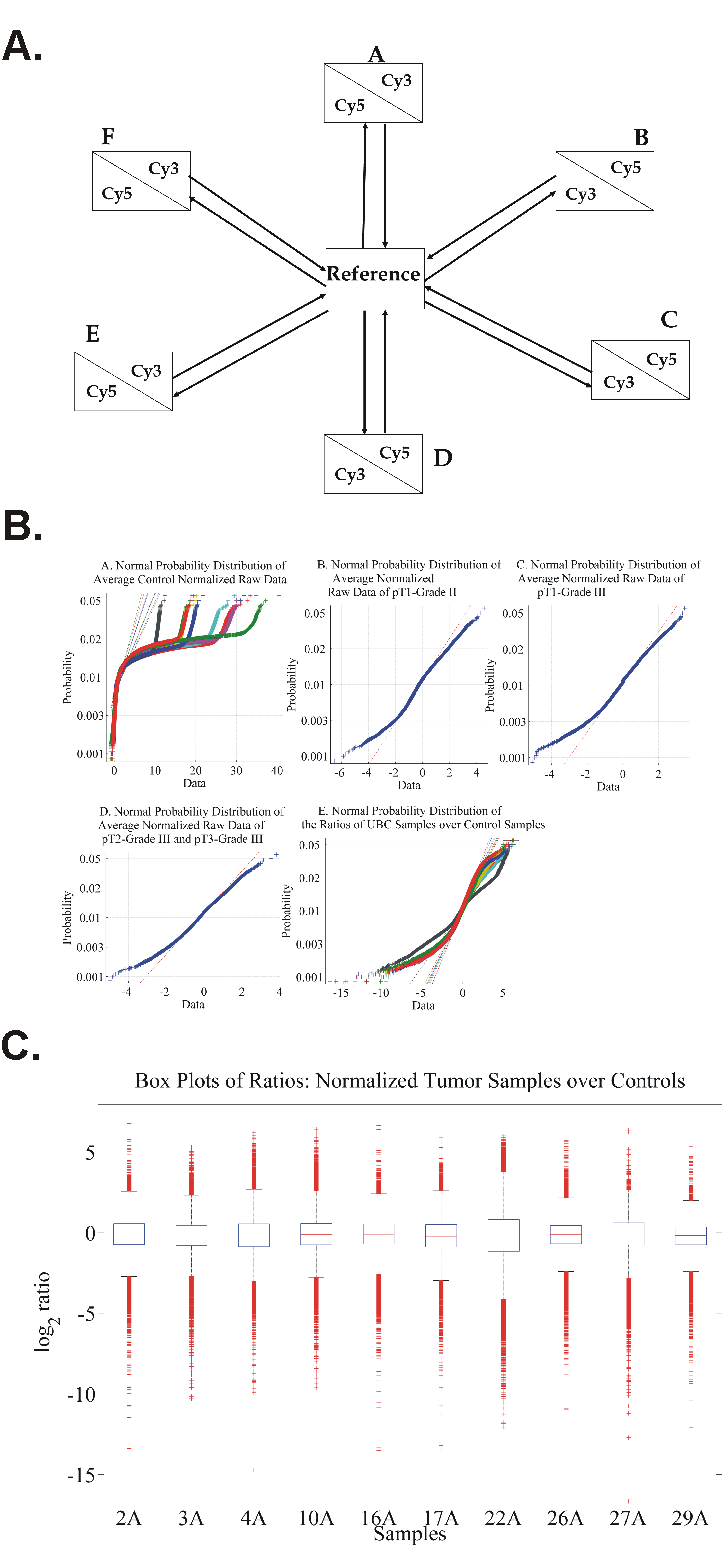

Supplement: Figure S1 — The reference-design was the experimental design of the present microarray experiments (A). Normal distributions of raw and normalized data (B). Box-plots of normalized log2-transformed ratios of samples against the average of controls (C). (TIF) [file pone.0018135.s001.tif]

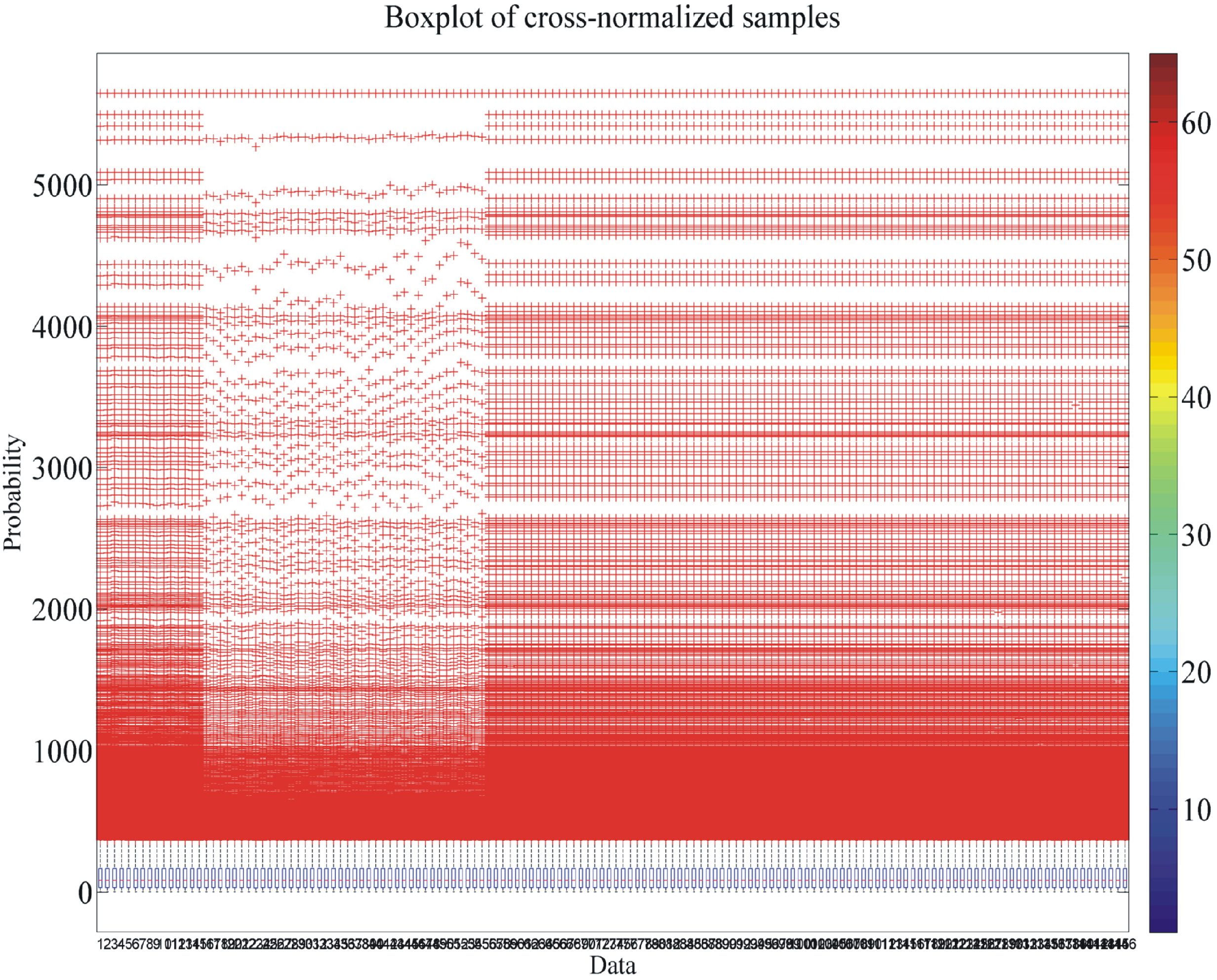

Supplement: Figure S2 — Results of cross-normalization between microarray samples. All samples were normalized each with the respective recommended platform normalization method. A further normalization followed using quantile normalization algorithm. (TIF) [file pone.0018135.s002.tif]

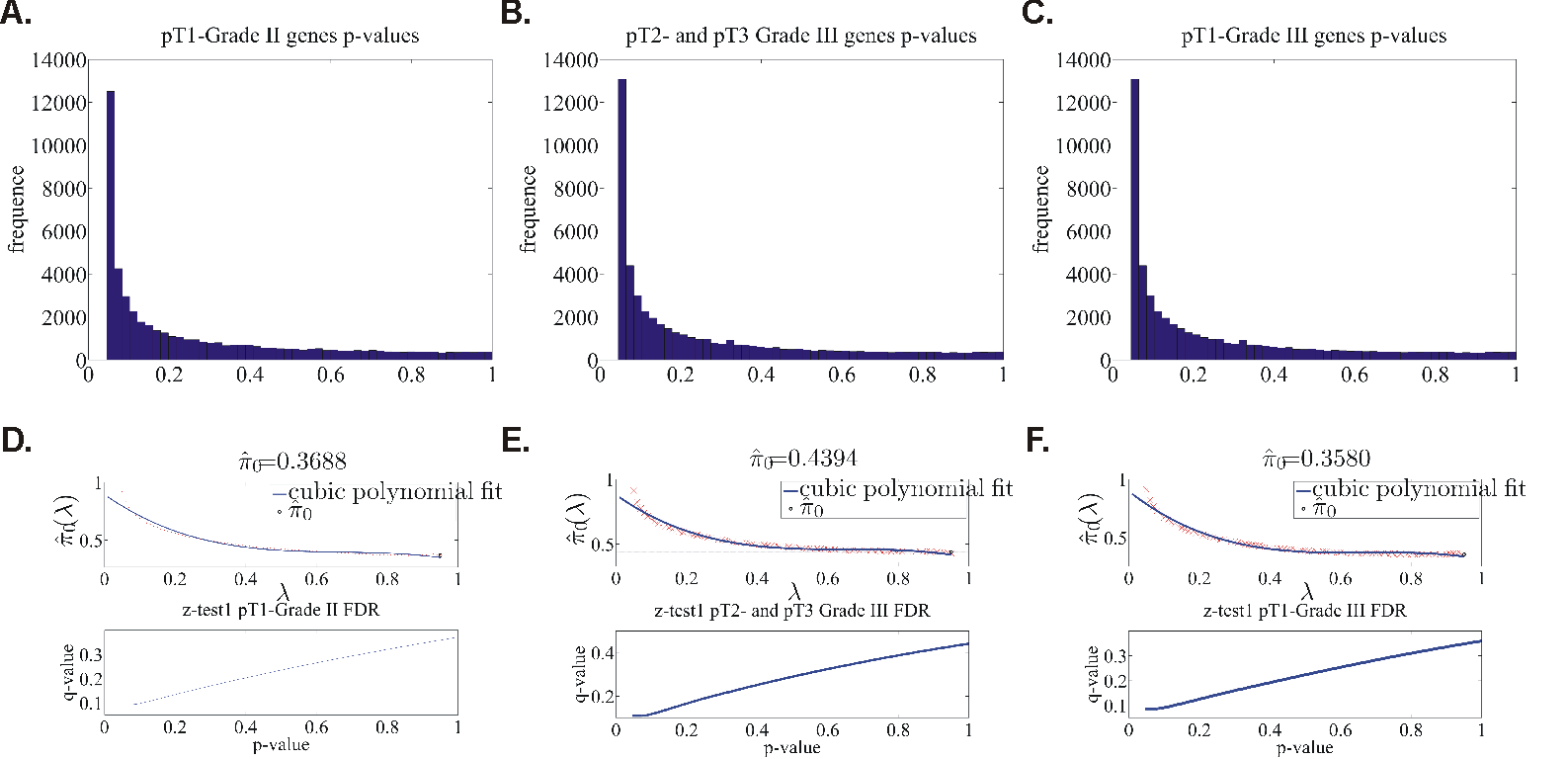

Supplement: Figure S3 — p -value Distribution of DE genes among tumor groups (A–C), along with FDRs (D–F). Genes that obtained a p-value<0.05 were considered as DEs. (TIF) [file pone.0018135.s003.tif]

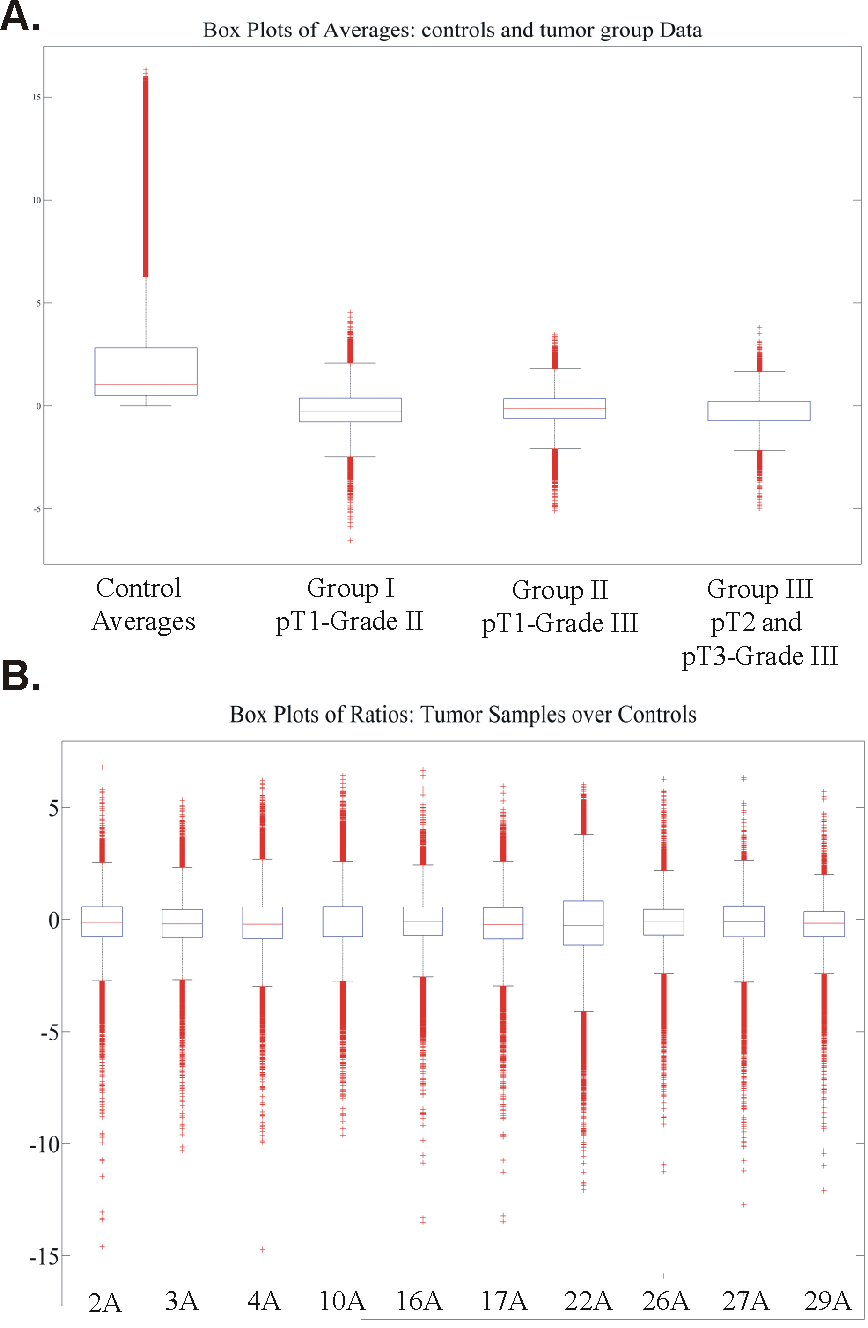

Supplement: Figure S4 — Box-plots of tumor groups (A) and individual samples (B). All samples in A and B with the exception of control averages are the log2 transformed ratios of samples over the control averages. (TIF) [file pone.0018135.s004.tif]

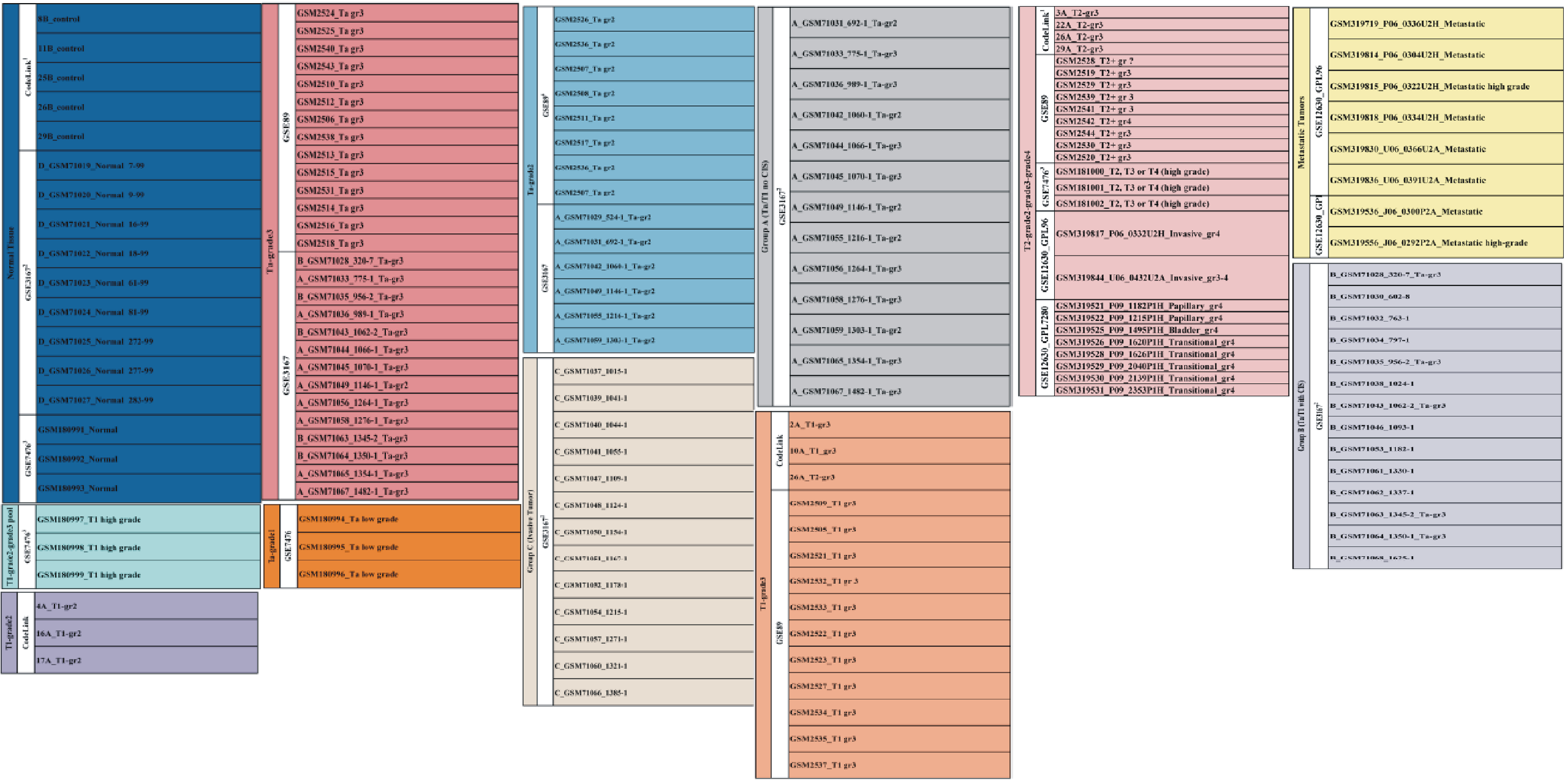

Supplement: Table S1 — The categorization of BC samples into groups is presented. (TIF) [file pone.0018135.s005.tif]

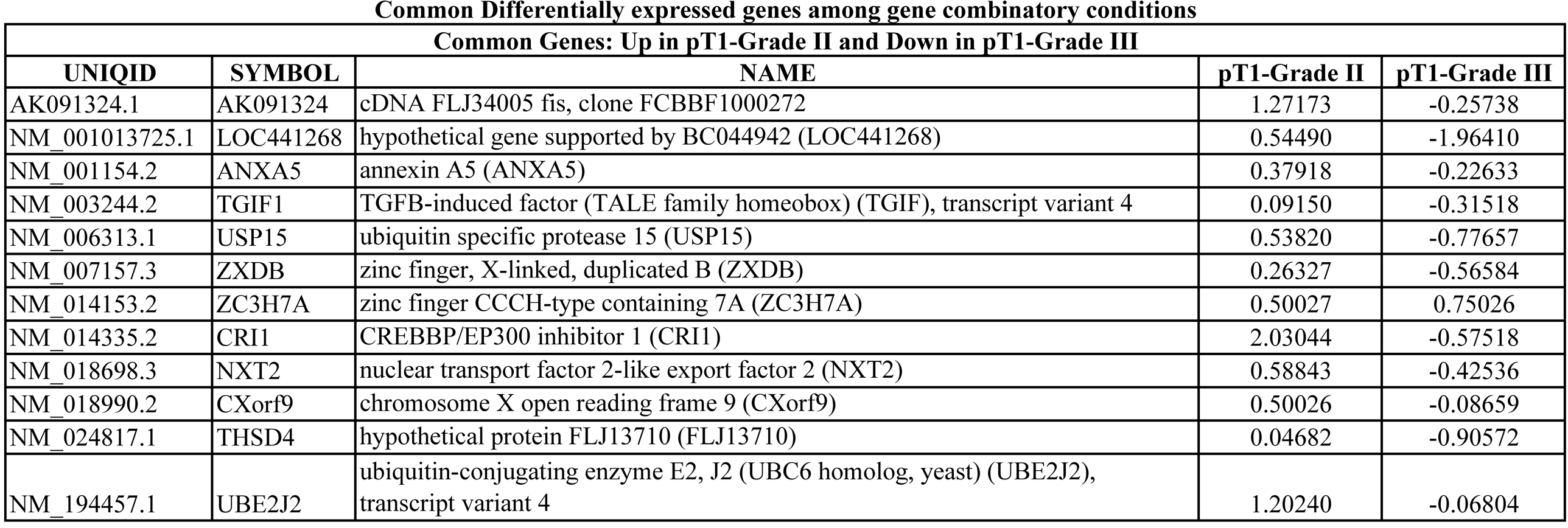

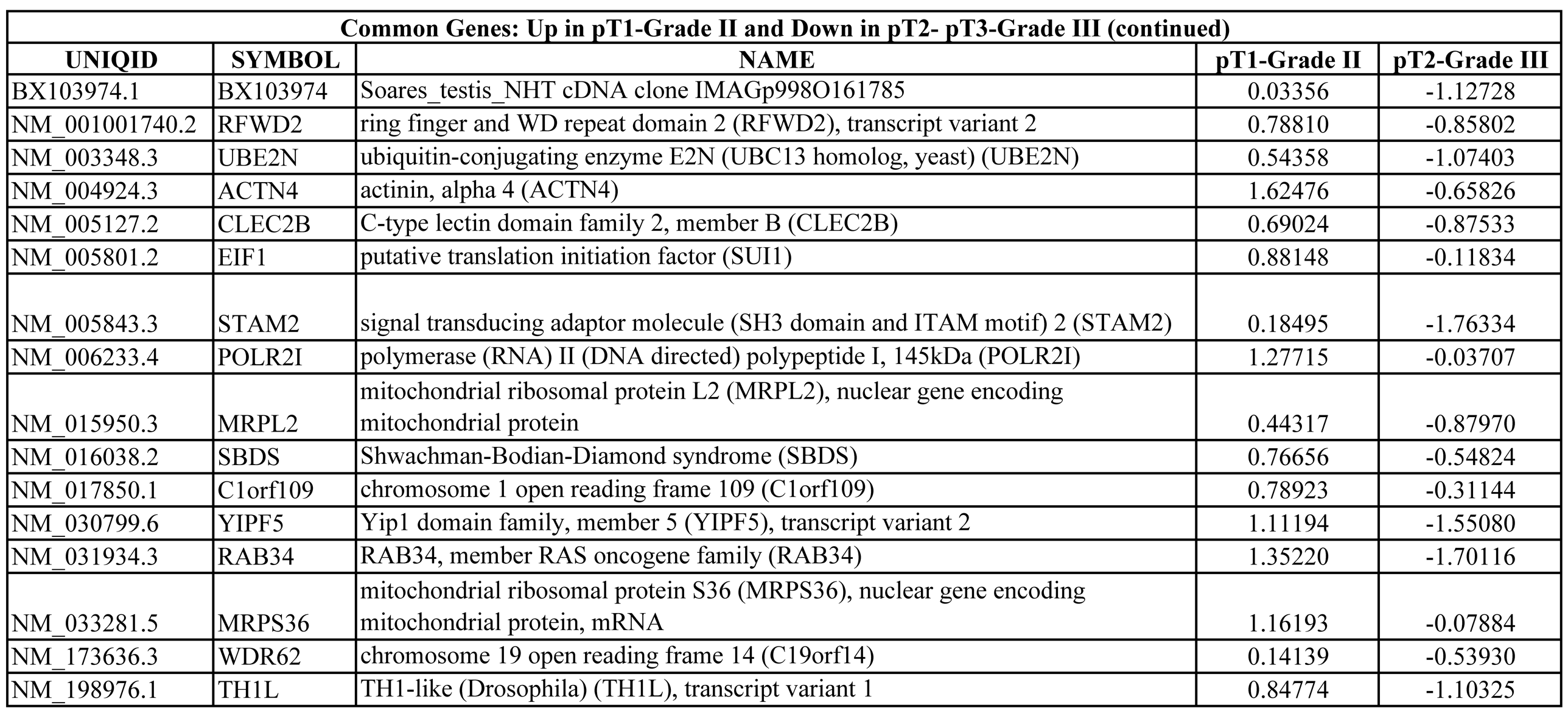

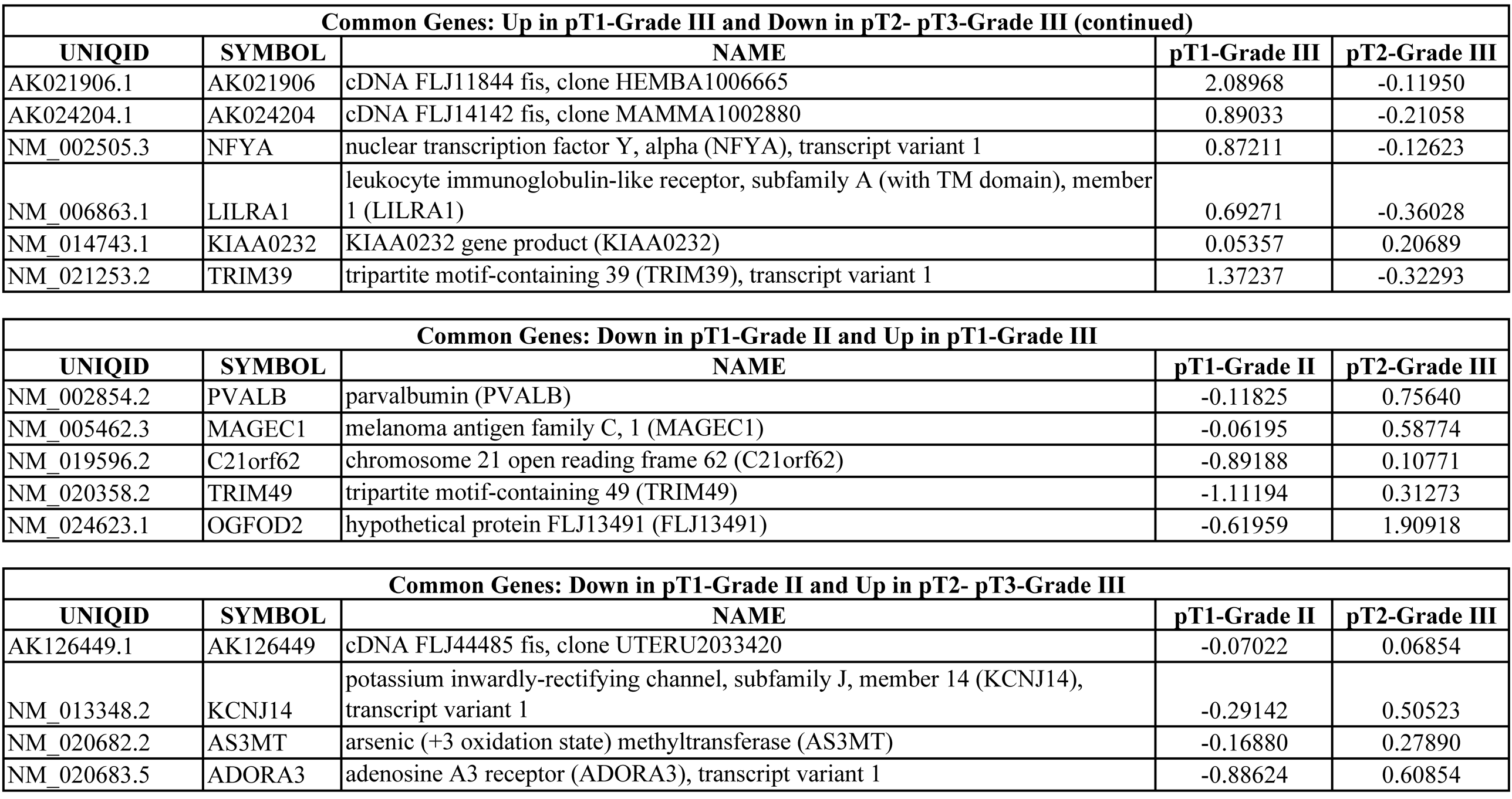

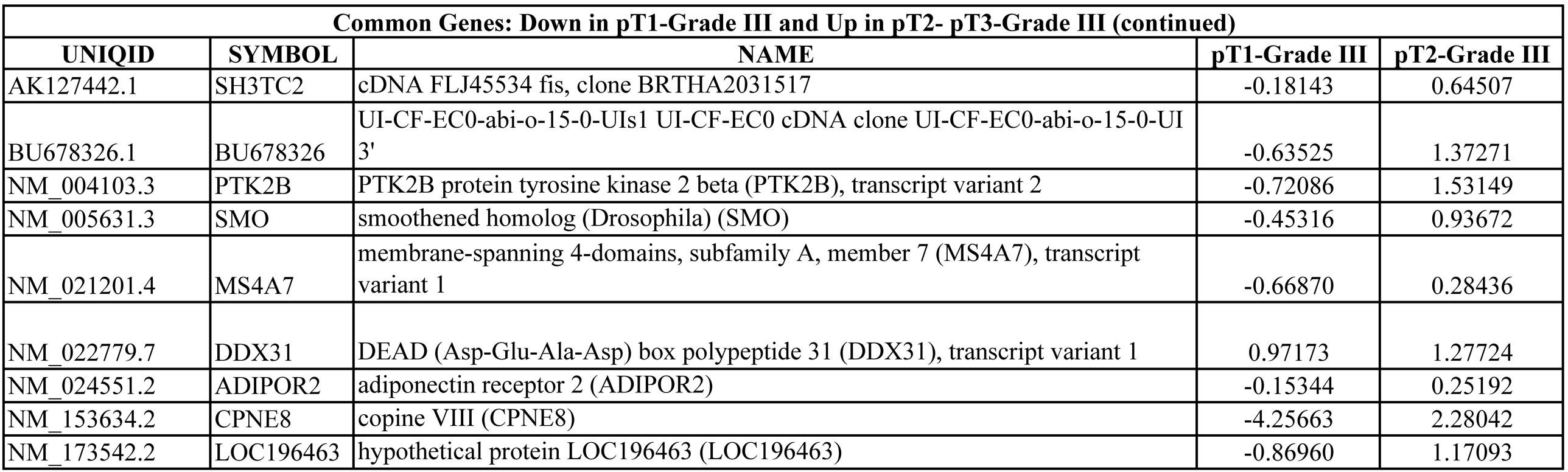


**Table S5**. Groups of common genes in several combinations.

Supplement: Table S5 — Groups of common genes in several combinations. (DOC) [file pone.0018135.s009.doc]
