# Supplementary material for: Identification of Common Differentially Expressed Genes in Urinary Bladder Cancer
Source: PLoS One. 2011 Apr 4;6(4):e18135. doi: 10.1371/journal.pone.0018135 (PMC3070717; doi:10.1371/journal.pone.0018135)
Supplement: Table S2 — Common Differentially Expressed genes between Groups pT1-Grade II (Group I) and pT1-Grade III (Group II) (p<0.05). (DOC) [file pone.0018135.s006.doc]

**Table S2**. Common Differentially Expressed Genes Between Groups pT1-Grade II (Group I) and pT1-Grade III (Group II) (p<0.05).
