# Supplementary material for: Identification of Common Differentially Expressed Genes in Urinary Bladder Cancer
Source: PLoS One. 2011 Apr 4;6(4):e18135. doi: 10.1371/journal.pone.0018135 (PMC3070717; doi:10.1371/journal.pone.0018135)
Supplement: Table S4 — Common Differentially Expressed Genes between groups pT1-Grade III (Group II) and pT2- pT3-Grade III (Group III) (p<0.05). (DOC) [file pone.0018135.s008.doc]

**Table S4.** Common Differentially Expressed Genes Between Groups pT1-Grade III (Group II) and pT2- pT3-Grade III (Group III) (p<0.05).
